# Improving the Process of Adjusting the Parameters of Finite Element Models of Healthy Human Intervertebral Discs by the Multi-Response Surface Method

**DOI:** 10.3390/ma10101116

**Published:** 2017-09-21

**Authors:** Fátima Somovilla Gómez, Rubén Lostado Lorza, Marina Corral Bobadilla, Rubén Escribano García

**Affiliations:** 1 Department of Mechanical Engineering, University of La Rioja, 26004 Logroño, La Rioja, Spain; fatima.somovilla@unirioja.es (F.S.G.); marina.corral@unirioja.es (M.C.B.); 2 IK4-LORTEK, 20240 Ordizia, Guipuzcoa, Spain; ruben.escribanogarcia@gmail.com

**Keywords:** Finite Elements Method, Multi Response Surface, optimization, biomechanics, human intervertebral lumbar disc

## Abstract

The kinematic behavior of models that are based on the finite element method (FEM) for modeling the human body depends greatly on an accurate estimate of the parameters that define such models. This task is complex, and any small difference between the actual biomaterial model and the simulation model based on FEM can be amplified enormously in the presence of nonlinearities. The current paper attempts to demonstrate how a combination of the FEM and the MRS methods with desirability functions can be used to obtain the material parameters that are most appropriate for use in defining the behavior of Finite Element (FE) models of the healthy human lumbar intervertebral disc (IVD). The FE model parameters were adjusted on the basis of experimental data from selected standard tests (compression, flexion, extension, shear, lateral bending, and torsion) and were developed as follows: First, three-dimensional parameterized FE models were generated on the basis of the mentioned standard tests. Then, 11 parameters were selected to define the proposed parameterized FE models. For each of the standard tests, regression models were generated using MRS to model the six stiffness and nine bulges of the healthy IVD models that were created by changing the parameters of the FE models. The optimal combination of the 11 parameters was based on three different adjustment criteria. The latter, in turn, were based on the combination of stiffness and bulges that were obtained from the standard test FE simulations. The first adjustment criteria considered stiffness and bulges to be equally important in the adjustment of FE model parameters. The second adjustment criteria considered stiffness as most important, whereas the third considered the bulges to be most important. The proposed adjustment methods were applied to a medium-sized human IVD that corresponded to the L3–L4 lumbar level with standard dimensions of width = 50 mm, depth = 35 mm, and height = 10 mm. Agreement between the kinematic behavior that was obtained with the optimized parameters and that obtained from the literature demonstrated that the proposed method is a powerful tool with which to adjust healthy IVD FE models when there are many parameters, stiffnesses, and bulges to which the models must adjust.

## 1. Introduction

The human intervertebral disc (IVD) is a fibrocartilage structure that is located between the vertebrae of the spine and absorbs the shock and pressure that are associated with daily movement. The healthy intervertebral disc provides mobility and spine flexibility during body movement and prevents excessive wear of the facet joints during daily movement of the spine. The behavior of the IVD is analogous to a ball full of water. The IVD is usually considered to be incompressible, similar to elastomers, due to its soft tissue and high water content. It permits limited motion while transmitting loads from one vertebra to another. Its complex structure permits significant mobility between adjacent vertebrae while transmitting considerable compressive loads. The IVD has three distinct regions [1]. These are the nucleus pulposus, the annulus fibrosus and the cartilage endplates. The endplate is composed of hyaline cartilage with a thin structure that covers the entire nucleus pulposus and about one third of the annulus fibrosus. The composition of the endplate is similar to a particular cartilage, but with a lower water content. The firm outer region, which is called annulus fibrosus, maintains the shape of the intervertebral disc. The annulus fibrosus is a complex ring structure that surrounds and protects the nucleus pulposus. It can be found between each pair of adjacent vertebrae. It is composed of concentric layers of fibrous tissue that have an approximate thickness of 1 mm. The fibers are an aggregate of collagen fibers and are alternatively oriented to the transverse plane at an angle of approximately ±30° [2]. The annulus fibrosus serves to resist the nucleus pressure in the radial and tangential directions [3]. The fibers are surrounded by a hydrated proteoglycam gel, or annulus ground substance [4]. The inner portion of the annulus is fibrocartilage, which gradually blends into the nucleus pulposus. The inner portion, which is called the nucleus pulposus, is the soft spongy tissue that enables the disc. It functions as a shock absorber, absorbing the impact of the body’s daily movements. The disc may be subjected to a complex combination of loads, but the nucleus pulposus helps to distribute the pressure evenly across the disc. This prevents the development of stress concentrations that could cause damage to the underlying vertebrae or their endplates. Many researchers have experimentally studied the behavior of the intervertebral disc of human cadavers by standardized tests (e.g., compression, flexion, extension, shear, torsion, and lateral bending) based on stiffness and disc bulges [5,6,7,8,9,10,11]. Today, due to medical ethics, the study of human cadavers is declining. Instead, many researchers carry out their studies with animals whose behavior is similar to that of humans [12]. Other researchers conduct their studies by modeling the behavior of the human body’s biomechanics by numerical methods (e.g., Finite Element Method, Finite Volume method, Finite-Difference method, etc.). FEM is known to be a powerful tool to design and optimize mechanical devices, as well as different human body parts or even human prostheses for foot, knee or hip. This is especially true when their behavior is nonlinear [13,14,15,16,17,18,19,20]. Over the years, several researchers have used the FEM to study the mechanical behavior of the human IVD. However, the parameters that define its complex structures differ entirely in each of these studies. Also, the process of achieving the best parameters to correctly define the behavior of the IVD FE models according to the standard test by trial-error method is difficult. This may result in an unacceptable cost. A large number of FE studies have proposed methodologies to adjust the deformations [21], the reaction forces [22] and the intradiscal pressure [23] of the proposed FE models were developed essentially by the trial-error method. However, an optimization algorithm was developed to determine the relationship among the components of the collagen fibers, annulus fibrosus and ground substance of an FE model using the biomaterial properties that were extracted from the literature [24]. In this case, the stiffness of the fibers was varied to approximate Young’s modulus of the ground substance in order to fulfil the required range of motions possible with the properties found in the literature. A method that was based on differential evolution was proposed by Ezquerro [25] to calibrate the FE model of a functional spinal unit that consisted of ligaments, nucleus pulposus, vertebral arch, and facet joints. In this case, the standard loads that were used were: flexion, extension, lateral bending and torsion. More recently, some researchers have used FEM and regression models based on support vector machine (SVM) combined with genetic algorithms (GA) to adjust the IVD FE model parameters [26]. Eleven material parameters were considered in this paper to define the behavior of the human IVD FE model. They were adjusted with nine different variations of stiffness and bulges from some of the previously mentioned standardized tests. These were two parameters for the nucleus pulposus (C10 and C0); two parameters for the cartilage endplate (E, μ); five parameters for the annulus fibers (Fiber12, Fiber34, Fiber56, Fiber78, and Fiber910), and two for the annulus ground substance (E, µ). The lowest value of error (around 6%) between the stiffness and bulges that were obtained from the proposed FE models and those obtained from the selected standardized test indicated that the proposed methodology was very effective. To date, no further references have been found in the literature for adjustment of parameters that define the non-linear behavior of human IVD FE models. However, some researchers have used the MRS method combined with FEM in order to study and optimize mechanical problems [27,28,29]. The current paper attempts to show how the combination of the FEM and the MRS methods with desirability functions can be used to obtain the most appropriate material parameters to use in defining the behavior of FE models of the human intervertebral lumbar disc. The proposed adjustment methods were applied to a medium-sized human IVD corresponding to the L3–L4 lumbar level with standard dimensions of width = 50 mm, depth = 35 mm, and height = 10 mm and developed as follows: First, a three-dimensional parameterized FE model that consisted of nucleus pulposus, cartilage endplate, annulus fibers, and annulus ground substance was developed and simulated with different standard tests. As in Somovilla Gómez et al. [26], and based on standard tests (e.g., compression, torsion, shear, flexion, extension, and lateral bending), three-dimensional parameterized FE models were generated that consisted of nucleus pulposus, cartilage endplate, annulus fibers, and annulus ground substance. Eleven parameters were used to define the parameterized FE models. Then, for each of the standard tests, regression models were generated to model six stiffness and nine bulges of the healthy IVD models as the parameters of the FE models were changed. Finally, an optimal combination of the eleven parameters was achieved by applying MRS based on desirability functions according to three different adjustment criteria. The first considered stiffness and bulges to be equally important for adjustment of FE model parameters. The second considered stiffness as most important, whereas the third considered bulges to be most important. An agreement between the kinematic behavior that was obtained with the optimized parameters and those obtained experimentally from the literature demonstrated that the proposed method is a powerful tool to use in adjusting healthy IVD-FE models when the latter have a high number of parameters, stiffness values, and bulges to which the models must adjust.

## 2. Use of FEM for Modeling the Lumbar Intervertebral Disc

A majority of the authors consider the nucleus pulposus to be an incompressible material. Its behavior has been modeled with FEM. It is considered to be a non-linear incompressible solid [30] as well as an incompressible fluid [11]. This non-linear behavior is due to its soft tissue condition and high water content [31,32]. In contrast, other authors have modeled the nucleus pulposus considering it to be a linear isotropic incompressible solid, with a Young’s modulus of 0.1 MPa [2,33,34,35], 1 MPa [36,37,38,39,40] and in a range of 0.5 to 1 MPa [41,42] or in a range of 1 to 4 MPa [43], whereas its Poisson’s module was 0.4999 or 0.5. Other authors have considered the incompressible elastomer formulation based on the Mooney-Rivlin model for modeling nucleus pulposus behavior. In this case, C10 and C0 are the empirical constants parameters that are most frequently used. For example, Smit et al. [41] considered the nucleus pulposus as incompressible and used the Mooney-Rivlin model with the parameters C10 = 0.12 and C0 = 0.09 and μ = 0.4999 to model it. Some authors also have considered the Mooney-Rivlin model with values of C10 = 0.12 and C0 = 0.03 [3,30,44], whereas others have used values of C10 = 0.10 and C0 = 0.09 [45,46]. Similarly, Ibarz et al. [47] considered parameters of C10 = 0.0343 MPa and C0 = 0.1369 MPa (E = 1.0 MPa and μ = 0.49).

On the other hand, Lu et al. [48], Martínez et al. [49], and González Gutiérrez et al. [42] considered the endplates’ cartilage behavior. They assumed an isotropic formulation with an elastic modulus E and a Poisson ratio μ of 20 MPa and 0.3, respectively. In a similar fashion, Kim et al. [36] and Dicko et al. [37], used the values for the endplates cartilage of E = 24 MPa and μ = 0.4, whereas Rohlmann et al. [46] and Schmidt et al. [44] used values of E = 23.8 MPa and μ = 0.8. However, Denozière [2] considered values of E = 12 MPa and μ = 0.3. The literature provides a Young’s modulus for the annulus ground substance of 2 MPa to 10 MPa. However, the most commonly used value is 4.2 MPa. Denozière [50], Dietrich et al. [51], and Baroud et al. [30] selected values of 4.2 and 10 MPa, respectively, for Young’s modulus. For Poisson’s ratio, they used values of 0.45 to 0.35. Other researchers considered the annulus ground substance as an incompressible, and used the Mooney-Rivlin model with the parameters of C10 = 0.348, C0 = 0.3, and μ = 0.45 [45,46,52]. Schmidt et al. [44] used values of C10 = 0.10, C0 = 0.05 and μ = 0.45. Dicko et al. [37] considered C10 = 0.18, C0 = 0.04, and μ = 0.45. Ayturk et al. [38] considered the annulus ground substance as a Yeoh hookean hyperelastic material that is a model for the deformation of nearly incompressible nonlinear realistic materials such a rubber. For the collagen fiber layers, most researchers have chosen linear properties in their studies, which provide an average Young’s modulus of approximately 360 to 550 MPa and a Poisson’ ratio of 0.3 to 0.45 [2,30,33,35,36,38]. However, Grauer et al. [39] considered values of 175 MPa for all fiber layers. Other authors assumed a nonlinear stress-strain behavior of the annulus collagen fiber layers based on a nonlinear function [37,44,45,46,52] that was obtained from previous reports that were based on the stress-strain curve of Shirazi-Adl et al. [11]. Ayturk et al. [38] considered the annulus fibers as Yeoh hyperelastic material with C10 = 0.0146, C20 = −0.0189, C30 = 0.04; a3 = 0.03, and b3 = 120. Table 1 shows the large number of parameters that some researchers have used during the last 30 years to develop intervertebral disc FE models. The latter adjusted the intervertebral disc FE models according to standard tests by trial and error, although this is difficult and very costly. To date, no author has made a methodical adjustment of these parameters. Only Somovilla Gómez et al. [26] have used FEM and Data Mining Techniques to adjust intervertebral disc FE models. In this paper, the authors propose the adjustment of the parameters that best define the behavior of the intervertebral disc. They propose the use of FEM and MRS with desirability functions and consider three different adjustment criteria. The first considers stiffness and bulges to be equally important, the second considers stiffness as most important, and the third considers bulges as most important.

### 2.1. Biomechanics of the Intervertebral Disc

**Figure 1:**
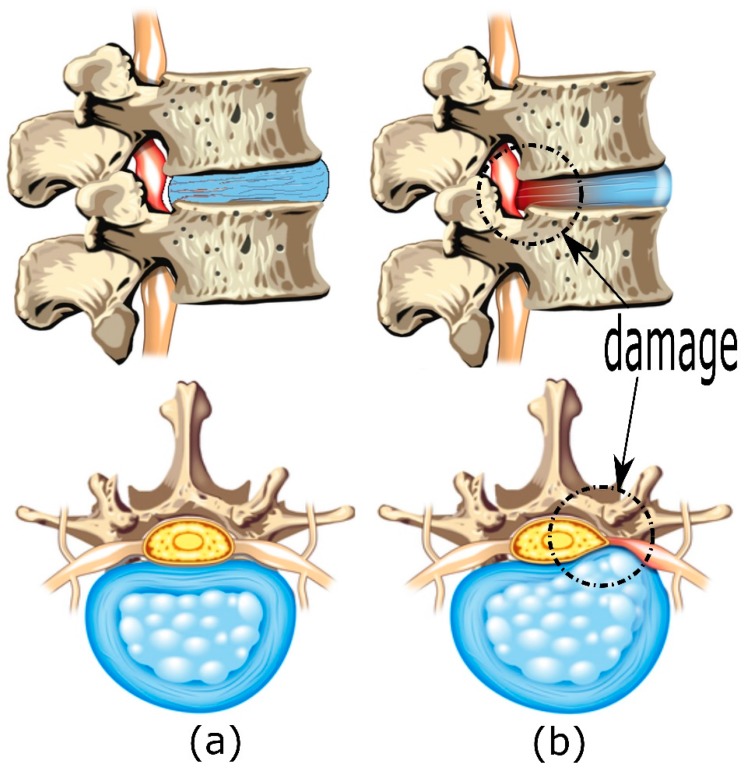
(**a**) Healthy Functional Spinal Unit (**b**) Detailed view of the nerve impingement and herniated intervertebral disc.

The intervertebral disc (IVD) is one of the most important constitutive elements of a functional spine unit (FSU). An FSU consists of two adjacent vertebrae, the intervertebral disc, and all adjoining ligaments between them. The FSL excludes other connecting tissues such as muscles. Its complex structure allows significant mobility between two adjacent vertebrae while transmitting considerable compressive loads from one vertebra to another. Over time, the normal aging process causes the intervertebral discs to degenerate. This reduces the water contained in the intervertebral disc and the ability to absorb the impacts that are associated with spinal movements. Excessive pressure, strain, or injury to a rigid disc can cause the disc to tear or bulge. The degeneration of the disc’s size and the loss of its functionality will bring the adjacent vertebrae closer to one another. This causes impingement and compression of a spinal nerve root. Nerve impingement can result in intermittent low back pain or leg pain, depending on the level of impingement [3]. This problem can be relieved by a surgical procedure called artificial disc replacement. In this procedure, the damaged disc is replaced by an artificial prosthesis (arthroplasty). Figure 1a,b show, respectively, an FSU with a healthy disc and an FSU with a herniated disc.

### 2.2. Spine Movement

**Figure 2:**
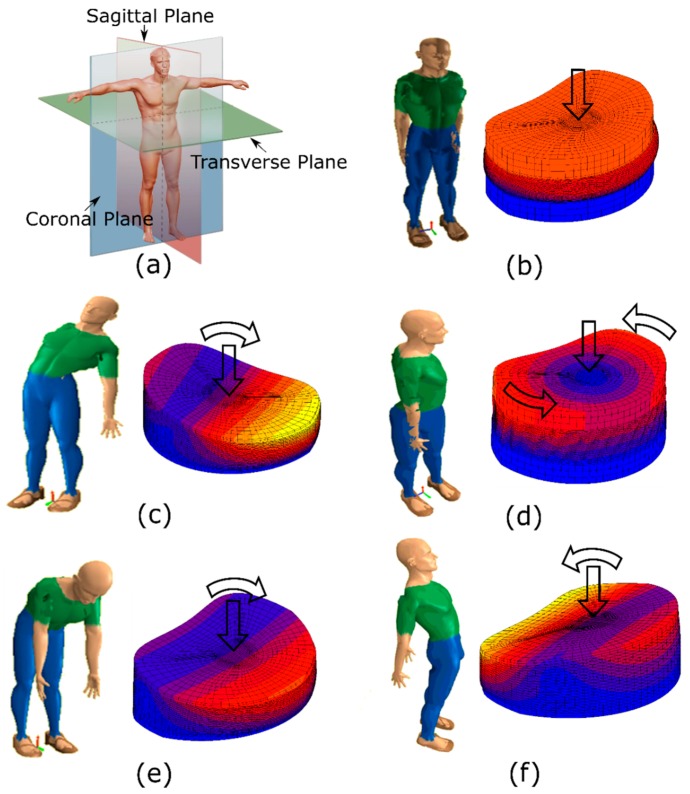
(**a**) Movement Planes; (**b**) Compression; (**c**) Lateral Bending; (**d**) Torsion; (**e**) Flexion; and (**f**) Extension.

Spinal movement during daily activities is complex because it involves a combination of plane movements with several motions in axial, coronal, and sagittal planes. In the coronal plane, the spinal movement takes place when an individual bends forward or backward. This typically occurs when one is exercising or performing heavy tasks. Forward bending of the spine is defined as flexion, whereas backward bending is called extension. Movement in the sagittal plane occurs when bending laterally to the right or to the left. Bending the spine to the right is called right bending and to the left is called left bending. Movement of the spine in the axial plane occurs after applying torsion clockwise and counterclockwise. Torsion of the spine is defined as torsion. Figure 2 shows the plane movements and the movements [53]: (a) Movement Planes; (b) Compression; (c) Lateral Bending; (d) Torsion; (e) Flexion; (f) Extension.

### 2.3. Bulge

**Figure 3:**
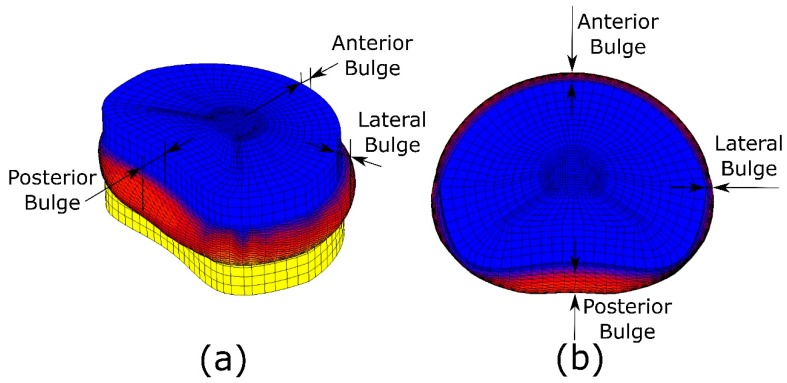
(**a**) Three-dimensional view of bulges and (**b**) actual dimension of the anterior, posterior, and lateral bulges.

In this work, the radial bulge of the lower disc was examined in the healthy model. The bulge was calculated at only three characteristic locations of the disc (anterior, lateral, and posterior) as shown in Figure 3, when axial compression, flexion, extension and lateral bending were simulated. The bulges for the torsion and shear tests were not considered. The disc bulged anteriorly during flexion, posteriorly during extension, and toward the concavity of the spinal curve during lateral bending. One of the mechanisms of nerve root irritation and herniated disc is root impingement by disc bulge. Clinical interest has led to several in vitro studies that have carefully measured the disc bulge. In the early studies, only the compression load was applied [54], but in later studies, the effects of various other loads also were considered in several directions, such as flexion, extension, and lateral bending [55,56,57,58,59].

### 2.4. Experimental Behavior of the Intervertebral Disc

For decades, most authors have studied the behavior of the human intervertebral disc by standard tests using human cadavers based on stiffness and disc bulges. The standard tests examine compression, flexion, extension, shear, lateral bending, and torsion. One of the first authors to study the behavior of the intervertebral disc was Virgin [60]. He investigated the elastic properties of the intervertebral disc in order to ascertain their contribution to the function of the vertebral column.

#### 2.4.1. Compression

In this work, Virgin [60] obtained a stiffness value of 2500 N/mm in a compression tests when he applied a load of 4500 N. Other authors used loads of 5500 N to 1000 N for the compression test and obtained stiffness values of 3000 to 700 N/mm [3,54,60,61,62,63,64]. In other works, for lower compression values with loads between 850 and 500 N, stiffness values between 2420 and 510 N/mm were obtained [5,7,65,66,67]. Others authors applied loads with values of less than 400 N and obtained stiffness values of 800 and 250 N/mm [8,68,69,70]. In regard to the bulges in the standard compression test, many authors analyzed the anterior, posterior, and lateral bulges of the intervertebral disc. In this regard, Shirazi-Adl et al. [11] used loads of 500, 720, and 1000 N and obtained values of 0.5–0.7–0.8 mm for the anterior bulge, 0.75–1.0–1.5 mm for the posterior bulge, and 0.35–0.4–0.6 mm for the lateral bulge. However, Denozière [2] considered a compression load of 2500 N and obtained results for the anterior, posterior and lateral bulges of 0.5, 0.7, and 0.4 mm. Table 2 summarizes the loads that were used in the compression test and the different stiffness and bulge values that were obtained by different authors.

#### 2.4.2. Flexion and Extension

One of the first studies of the behavior of Flexion and Extension of the human intervertebral disc was [61]. In this work, a load of 20 Nm was considered for both flexion and extension. Stiffness values of 4.5 Nm/° were obtained in both tests. Similarly, Brown et al. [69] considered the same load (20 Nm) but obtained a stiffness value of 2 Nm/° in both tests. Another study, Miller et al. [71], used higher loads (70 Nm), and obtained stiffness values of 5.51 and 7.60 Nm/° for the Flexion and Extension tests respectively. Finally, Adams et al. [72] used a load of 80 Nm and obtained stiffness values of 7.3 Nm/° for both tests. However, most researchers have used loads of 5 Nm to 10 Nm for their studies. For example, the work by Schultz et al. [9] considered a load of 10.6 Nm for a study of the behavior of the intervertebral disc, and obtained stiffness values of 1.92 Nm/° and 3.53 Nm/° for the Flexion and Extension test respectively. Adams et al. [73] considered a load of 10.7 Nm, and the stiffness obtained was 1.34 Nm/°. Other authors used a load of 10 Nm for the Flexion and Extension test. They obtained stiffness values that ranged between 0.8 and 2.04 Nm/° for Flexion, and between 2 and 3.53 Nm/° for Extension [7,53,67]. Guan et al. [74], Busscher et al. [75] and [76], González Gutiérrez [3], and Patwardhan et al. [77] considered a load range of 4 to 8 Nm for both the Flexion and Extension tests. The stiffness values that they obtained from these tests varied from 0.8 to 1.18 Nm/° for the Flexion test and from 0.8 to 1.53 Nm/° for the Extension test. In regard to the study of anterior, posterior and lateral bulges by the Flexion and Extension tests, Reuber et al. [58] considered loads of 3.9 Nm to 7.9 Nm and obtained values of 0.73 and 1.11 mm for the posterior bulge, respectively. For the lateral bulge, they obtained values of 0.07 and 0.21 mm respectively. Finally, Denozière [2] used a load of 10 Nm and obtained values of 1.3, 1.9, and 2.6 mm for the anterior, posterior, and lateral bulges, respectively. Table 3 summarizes the stiffness and bulge values for the Flexion and Extension standard tests by the various authors.

#### 2.4.3. Lateral Bending

As in the previous standard test, Schultz et al. [61] obtained a value of stiffness equal to 2.8 Nm/° for Lateral Bending tests using a load of 20 Nm. Subsequently, Miller et al. [71] obtained a stiffness value of 4.35 Nm/° for Lateral Bending using a load of 60 Nm. Other authors considered a lower range of loads (between 4 and 10 Nm) and obtained values that ranged between 0.75 and 2 Nm/° [3,7,9,53,74,75,76,78]. In addition, one of the few authors who were found in the extensive literature to have studied the anterior, posterior and lateral IVD bulges under a Lateral Bending load was Reuber, M. et al. [58]. In this work, two different loads of 9.8 Nm and 3.9 Nm were used. They resulted in bulge values of 0.49 and 1.13 mm for the posterior bulge respectively, whereas the lateral bulge values were 0.83 and 2.11 mm respectively (See Table 4).

#### 2.4.4. Shear and Torsion

Only a few studies in the literature have studied the stiffness of the IVD in a shear test. One of these was conducted by Schultz et al. in [9,61], who used loads of 1000 N and 980 N and obtained, respectively, 685 N/m and 1000 N/m for stiffness values. Similarly, Weisse et al. [79] obtained a stiffness value of 830 N/m when they applied a load of 950 N. Another research team to conduct this type of test was Liu et al. [6]. In this work, the load applied was 450 N and the stiffness value that was obtained was 300 N/m. Other authors, such as Miller et al. [71] and Markolf [62], applied loads of 150 N for the same shear test but obtained different stiffness values (115 N/m and 260 N/m). Finally, Moroney et al. [68] used a much lower load than those used by the previously mentioned authors (20 N) and obtained a stiffness value of 60 N/m.

When the disc is subjected to a torsion load, the stress distribution in the IVD depends on the disc’s degree of degeneration and whether the posterior elements are intact. Miller et al. [71] was one of the groups of investigators in the literature, who considered the highest torsion load. It was 70 Nm and produced a stiffness value of 10.9 Nm/°. Farfan et al. [80] and Schultz et al. [61] considered loads of 31 Nm and 30 Nm, and obtained stiffness values of 2 and 4.5 Nm/° respectively. However, most researchers considered torsion test loads of 10 Nm to 8 Nm and obtained stiffness values that ranged from 8.48 to 1.44 Nm/° [7,53,78,81,82]. Today, loads of 5 Nm to 4 Nm are applied in this type of standard test, and the stiffness values that are obtained vary from 4.4 to 1.6 Nm/° [42,75,76]. Table 5 summarizes the stiffness values and the loads applied in shear and torsion tests.

As described in the previous paragraphs, the published studies of the behavior of IVD are extensive. In this regard, the current work selected only fifteen different stiffness values and bulges, as well as the corresponding loads, for a search for the optimal combination of the disc’s FE model parameters. Table 6 summarizes the 15 different stiffness and bulge values that were selected (six for stiffness and nine for bulges) and the load that each author, whose work was selected, applied in the corresponding standard test. These values were used to adjust the parameters that define the behavior of the FE model.

## 3. FE Model for Modeling the Intervertebral Disc Proposed

**Figure 4:**
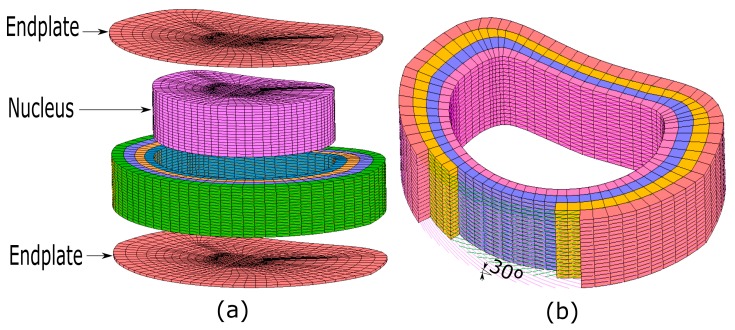
(**a**) Details of the FE model that is formed by the nucleus cartilage endplates, nucleus pulposus, and annulus fibrosus; and (**b**) details of the orientation of the five different fiber layers.

Like the authors’ disc that was modeled by FEM and described in Section 2 (See Table 1), this paper considered that intervertebral discs have four main parts. They are the annulus fibrosus, the nucleus pulposus, and the two endplates that link it to the vertebrae. Figure 4 shows a 3D view of the proposed human healthy IVD FE model. In the lumbar spine, the nucleus is located between the middle and the posterior third of the sagittal diameter. Its volume is 30–50% of the total disc volume [53]. In some finite element analyses of the lumbar spine, the authors concluded that the nucleus was 43% of the total disc volume, which is the volume that the current paper assumes.

Also, the nucleus pulposus was considered to be a non-linear incompressible and hyper-elastic Mooney Rivlin solid material. It was formulated according to empirical constants C10 and C0 [30,41]. It was assumed that the nucleus pulposus is surrounded by the annulus fibrosus. In order to prevent volumetric blockage, as the nucleus was considered to be practically incompressible, a full integration with Herrmann formulation was used for the eight-node solid FE elements that constituted the nucleus pulposus [83,84]. The annulus fibrosus was assumed to be a homogenous ground substance of hydrated proteoglycan gel that is reinforced by collagen fibers. The current FE model includes five layers of fibers that were oriented at an angle of ±30° relative to the transverse plane. The fibers were simulated by three-dimensional unidirectional line FE elements. The homogenous ground substance was simulated as eight-node isoparametric solid FE elements. For the five layers (Fiber12, Fiber34, Fiber56, Fiber78, and Fiber910), a range of E of 550 to 360 MPa, respectively, was assumed for their elastic modulus values. The endplates, which are composed of hyaline cartilage, link the disc to the vertebrae. This work considered cartilage endplates with an isotropic formulation (an elastic modulus E and a Poisson ratio of μ) for the FE model). A total of 11 material parameters were selected to define the behavior of the human disc FE model. They were adjusted after considering the above mentioned standard test. Table 7 summarizes these eleven different proposed material parameters.

### 3.1. Configuration of the Finite Element Analysis and Mesh Size

**Figure 5:**
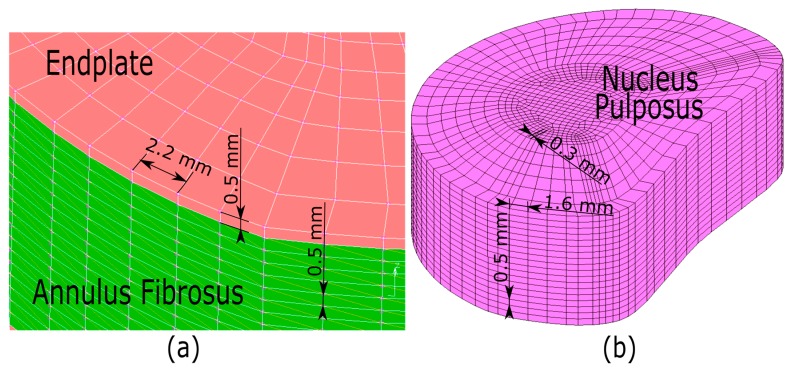
(**a**) Details of the mesh size for the endplate and annulus fibrosus; and (**b**) details of the mesh size for the nucleus pulposus.

Due to the large displacements and deformations that the intervertebral disc suffered in this study, as well as the hyper-elastic behavior of the nucleus pulposus, a nonlinear analysis that involved large strain procedure with an updated Lagrangian formulation was used. Also, the mesh size that was established for each of the four parts of the intervertebral disc was based on the extensive literature available [85,86]. In this case, the largest mesh size for hexahedral elements of the endplate and annulus fibrosus was 2.2 mm, whereas the lowest was 0.5 mm, which corresponds to the thickness of the endplate itself (See Figure 5a). Also, the largest mesh size for the nucleus pulposus was 1.6 mm, and the smallest size was 0.3 mm (See Figure 5b). The openings in all of the mesh sizes that were established for the proposed FE models were smaller than each of the four parts of the intervertebral disc than those sizes of the FE models proposed in the literature. In other words, the discretization that is chosen can obtain satisfactory results without depending on further mesh refinement.

### 3.2. Intervertebral Disc Dimensions

**Figure 6:**
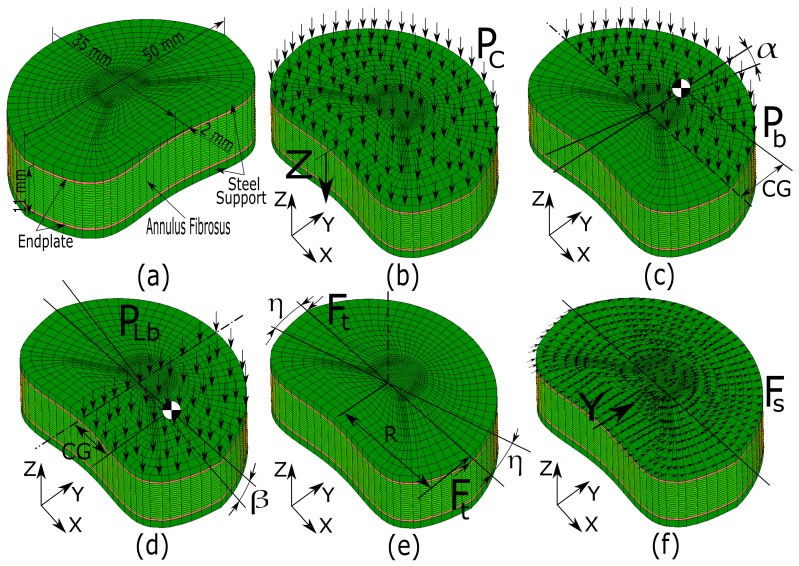
Intervertebral disc (IVD) dimensions and boundary conditions necessary for the standard test: (**a**) IVD dimensions; (**b**) compression load; (**c**) flexion load; (**d**) lateral bending load; (**e**) torsion load; and (**f**) shear load.

For decades, many authors have studied the kinematic behavior of human intervertebral discs of patients of different ages, sex, and stature, as well as different states of degeneration of the intervertebral disc itself. Many of these studies have been based exclusively on experimental studies using intervertebral discs from cadavers. Other works have been based mainly on FE models of discs for the study of their kinematics. In the studies that were based on FE models, the proposed models have been experimentally validated using data obtained by the authors themselves, or have been experimentally validated using data obtained from the literature. For a very broad range of patients that were studied, all lumbar intervertebral discs for the L1–L5 segment analyzed have had very similar widths, depths, and heights (See Figure 6a). For example, Zhou et al. [87] studied 126 patients with differing degrees of disc degeneration according to the Friberg and Hirsch scale [88]. Fifty-five male patients with a mean age of 50 ± 13.6 and 71 female patients with a mean age of 49 ± 12.04 and an age range of 22–80 years were studied. The approximate dimensions of the intervertebral discs analyzed in this case were, according to Figure 6a, a width of 46.1 ± 3.1 mm, a depth of 34.1 ± 2.6 mm, and a height of 12.4 ± 1.7 mm. Rostedt et al. [5] experimentally studied six lumbar FSUs from four subjects with different degrees of disc degeneration. The less degenerate discs (with a degree of degeneration equal to 1) were those of individuals of 45 years of age and a disc height of 12 mm. Schultz et al. [9] studied 16 UVF from 10 females and 26 UVF from 13 males with an age range of 21 to 60 and a mean age of 43 years with different degrees of degeneration. In this case, the less damaged discs were those of men of 35, 40, and 53 years of age with a disc surface of 15.9, 16.8, and 15 cm^2^, respectively. Other authors, such as Panjabi [89], studied UVF with healthy intervertebral discs from a sample of 60 patients of age 19–59 years (a mean of 46.3) and disc dimensions of width of 48.1 mm and a depth of 34.7 mm. Eijkelkamp [90] also studied a group of 60 patients of age 18 to 65 years, and observed that the mean intervertebral disc height was 13.5 mm. With a group of 157 patients and ages between 20 and 38 years, Nissan and Gilad [91] determined an average depth and height of the disc of 34.6 mm and 10.8 mm, respectively. With a smaller group (11 individuals) of age between 25 and 36 years, Tibrewal and Pearcy [92] found a depth and height of the disc of 33 mm and 9.8 mm, respectively. However, Wolf et al. [93] determined a mean width and depth of 44.1 mm and 31.7 mm respectively for a disc in a group of 55 individuals of 20 to 90 years of age. A more extensive study was that of Amonoo-Kuofi [94]. In the latter case, a group of 305 males and 310 females of 10 to 64 years of age and 10 to 61 years respectively were studied. The mean dimensions of the intervertebral discs were a depth of 42.8 mm and a height of 13.5 mm for males and a depth of 39.9 mm and a height of 13 mm for females. Other authors proposed FE models and validated them later with their own experimental data. For example, Schmidt et al. [24] developed a method to determine the individual contribution of the fibers and the ground substance to bending moments with four different magnitudes. They proposed a 3D, non-linear FE-model of an intervertebral disc from the L4–L5 FSU. In order to determine the appropriate geometry of the FE-model, six specimens were tested. The median anteroposterior dimension of the inferior endplate of L4 was 37.4 mm, whereas the median left to right dimension was 58.7 mm. The geometry used in the FE-model was determined from one of the tested specimens. The latter had dimensions nearest to the median with an anteroposterior distance of 38.1 mm and a left to right distance of 59.0 mm. Kim et al. [36] studied with the FEM the influence of the disc degeneration. A 3D nonlinear FE model of the lumbar spine was developed that consisted of four lumbar vertebrae, three intervertebral discs, and the associated spinal ligaments. Geometrical details of the human lumbar spine (L2–L5) were obtained from the high-resolution computed tomography (CT) images of a 46-year-old male patient who had no spinal deformities. In this case, the average area of the intervertebral disc was 1119 mm^2^. González et al. [42] studied the biomechanics of the intervertebral disc with different degrees of degeneration in compression using a FE model that was validated by experimental data. The study was performed on 5 patients aged 65–75 years for L2–L3 and L4–L5 lumbar levels. The height of the healthy intervertebral disc that was studied was 9.9 mm with an area of 1845 mm^2^ for the L2–L3 level. For the L4–L5 level, it was 10 mm with an area of 1951 mm^2^. Shirazi-Adl et al. [11] proposed an FE model for analyzing the stress of the UVF para el nivel lumbar L2–L3 in compression for a 29 years old female patient. The geometry of the FE model that was analyzed was based in in vitro measurements. It had a width of 49.2 mm, a depth of 34 mm, a heigth of 11 mm and an area of 1371 mm^2^. Smit et al. [41] proposed a complete UVF FE model and studied the stresses on the cortical bone of the vertebrae. In that case, the intervertebral disc that was proposed in the FE model had a width of 42 mm and a depth of 35 mm. Finally, other authors proposed FE models and later validated them with experimental data from other authors. For example, Ibarz et al. [47] proposed a FE model for the Lumbar Spine to study the degeneration of the intervertebral discs. To verify the FE model, radiological images (X-rays) were taken of a group of 25 healthy male individuals who had an average age of 27.4 and average weight of 78.6 kg. The results obtained from the simulation and from radiological measurements were compared to the results obtained from White and Panjabi [95]. Good agreement was obtained for the different movements. However, Ayturk [38] proposed an FE model to study the changes in lumbar spine mechanics due to degenerative disc disease. The experimental study was conducted on a 49-year-old female patient. The results obtained from the FE model were also compared to the studies that were conducted by Heuer et al. [96]. Other authors such as Weisse et al. [79] proposed an FE model for the determination of the translational and rotational stiffness of an L4–L5 FSU using the FEM. In this case, the geometry of the disc was obtained by computed tomography, and the results were compared subsequently to the studies by several of the aforementioned authors [87,89,90,91,92,93,94]. Once the proposed FE model was simulated, the results were compared experimentally to results obtained from the literature [96,97,98]. Denozière and Ku [50] proposed a 3D FE model of a two-level ligamentous lumbar segment. The proposed FE model was validated with the clinical data obtained from the literature [98,99,100,101]. These authors developed an FE model of the intervertebral disc according to Figure 6a. In this case, the intervertebral disc had a width of 50 mm, a depth of 35 mm, a height of 10 mm and an area of 1440 mm^2^. Table 8 summarizes the anatomical dimensions of the L1–L5 lumbar segment and the differents intervertebral discs that were studied. As has been observed from the references in Table 8, the dimensions of the intervertebral discs are very similar for the range of patients studied. Also, it can be seen in this table that many of the mentioned authors validate their FE models with the experimental data obtained from the studies by other authors. For this reason, the FE model of the intervertebral disc that is studied in the current paper has been elaborated on the basis of the study by Denozière and Ku [50], which had the following dimensions according to Figure 6a (width = 50 mm, depth = 35 mm, height = 10 mm, and area = 1440 mm^2^).

### 3.3. Boundary Conditions

Numerous authors have studied the behavior of intervertebral discs with the FEM. In doing do, they applied the contour conditions to these models and thus reproduced, as rigorously as possible, the corresponding experimental analysis [2,3,50]. In the current paper, the boundary conditions applied to the proposed FE models were applied in a similar way to the work that was developed by Denozière [2] and Denozière and Ku [50]. The proposed intervertebral disc FE model consisted of the intervertebral disc itself (nucleus pulposus, endplate, annulus fibrosus, and the fiber layers), as well as two steel supports to which the disc was glued (See Figure 6a). The boundary conditions applied to the proposed FE model to obtain the compression stiffness were applied as follows (see Figure 6b). On the upper steel support the pressure P_c_ was applied. It was calculated by the following equation:

(1) $$P_{c}=\frac{F_{\max}}{S}$$

where F_max_ is the maximum compression test load, whose value was 500 N (See Table 6). S is the upper surface of the steel support, which has an area of 1440 mm^2^. The compression stiffness value was obtained from the “Z” displacement. The boundary conditions to obtain the stiffness value for the bending test were applied as follows (See Figure 6c): the corresponding maximum torque of the bending test (B_max_) was applied on the anterior area of the steel upper support, which, in this case, is 5 Nm (See Table 6). The pressure P_b_ was calculated as follows:

(2) $$P_{b}=\frac{B_{\max}}{S_{a}\cdot \mathrm{CG}}$$

where S_a_ is the anterior area of the intervertebral disc, namely 745 mm^2^, and CG is the distance from the geometric center of the disc surface or instantaneous axis of rotation (IAR) to the center of gravity of S_a_. The bending stiffness value was calculated once the angle α had been obtained. The boundary conditions of the FE model that were required to obtain the lateral bending stiffness were applied in similar fashion to the process to obtain the bending stiffness (see Figure 6d). Over half area of the upper steel support was applied the pressure P_lb_, which was calculated according to the following equation:

(3) $$P_{\mathrm{lb}}=\frac{{\mathrm{LB}}_{\max}}{(S/2)\cdot \mathrm{CG}}$$

where LB_max_ is the maximum torque of the bending test, which in this case was to 10.6 Nm (see Table 6). S/2 is the area of the intervertebral disc that is considered for the lateral bending, namely 720 mm^2^ and CG is the distance from the geometric center of the disc to the center of gravity of S/2 area. The lateral bending stiffness was obtained from angle β. The boundary conditions necessary to obtain the torsional stiffness (see Figure 6e) are: on a group of nodes that belong to the periphery of the steel upper plate and a force F_t_ (in this case in the Y direction) that is applied in a way that twists the disc. In order that the load F_t_ is always at an angle of 90° to a given R, a “following force” analysis was considered. The value of this force F_t_ was calculated with the following equation:

(4) $$F_{t}=\frac{T_{\max}}{n\cdot R}$$

where T_max_ is the maximum torque value. In this case, it is 10 Nm (see Table 6), Also, n is the number of nodes at which the load F_t_ was applied and R is one the half of the width of the intervertebral disc (in this case is 25 mm). The value of the stiffness of lateral flexion is obtained from the angle η. Finally, the boundary conditions that are necessary to obtain shear stiffness (see Figure 6f) are as follows: on all of the nodes on the upper surface of the steel support, an F_s_ load was applied in the Y direction. The value of this force F_s_ was calculated with the following equation:

(5) $$F_{s}=\frac{F_{\max}}{n}$$

where F_max_ is the value of the maximum torque, which is 450 N in this case (see Table 6), and n is the number of nodes to which the load F_s_ is applied. The value of the stiffness of shear was obtained from the Y displacement of these nodes. In addition, for all previously proposed simulations, a boundary condition of embedding all nodes was imposed on the lower support of steel.

## 4. Design of the Experiments and Design Matrix

The response surface method (RSM) needs to establish a design of experiments (DoE) in order to obtain accurate models with the least amount of data to support the initial hypotheses [102]. Several methods have been proposed to develop DoE. However, they generally require the construction of a design matrix (inputs) to measure the outputs or responses of the experiments. One of the most widely used methods is the full factorial design method [103]. This method is based on the adoption all possible combinations of each of the values (or levels) and each of the factors that are considered in the DoE. Another DoE that is widely used is the 2k factorial design. This DoE is a full factorial design that has two levels and generates 2k experiments in which k is the number of factors. In using this method to adjust the parameters of the FE model, the number of factors is k = 11 (C0, C10, Fiber12, Fiber34, Fiber56, Fiber78, Fiber910, Annulus_E, Annulus_μ, Cartil_E, and Cartil_μ) and the number of experiments or FE simulations needed is 2048. Similarly, a 3k factorial design generates 3k experiments or 177147 FE simulations. For both methods, this amount of data may be sufficient to cover completely the entire range of possibilities. However, it has the disadvantage of generating a large number of experiments or FE simulations. Another of the methods that are used to develop a DoE is Central Composite Design (CCD). This method is considered to be a fractional three-level design that is useful in obtaining regression models, thereby reducing the number of experiments in comparison to a factorial design 3k. A CCD generates 2070 experiments. This is very similar to the number that a 2k factorial generates. However, a Box-Behnken design (BBD) generates 176 experiments. This is an advantage because it significantly reduces the number of FE simulations [104]. In the current paper, the DoE was employed using a two-level fractional factorial design. In this case, 128 FE simulations were needed. This reduced amount of data may be sufficient to completely cover the entire range of possibilities and subsequently to search for the optimal parameters that define the behavior of the intervertebral disc. Table 9 shows some of the 128 combinations of the material parameters (C0, C10, Fiber 12, AnnulusE, etc.) that are generated with a two-level fractional factorial design when the ranges that appear in Table 7 are considered. The design matrix and their corresponding combination of parameters that define de behavior of the human lumbar Intervertebral disc FE models are generated by using the “R” statistical software (R 2014).

### 4.1. Response Surface Method for Modeling and Optimizing Problems

RSM is a method that attempts to determine the relationships between input variables and one or more response variables. The method was introduced by Box and Wilson in [104] and was used first to obtain a regression model from experimental data and an optimal response. Originally, RSM was developed to model experimental responses. However, it has been used recently to optimize products and industrial processes [105,106]. RSM is a group of statistical techniques that utilize a regression model that is based on a polynomial function (Equation (6)).

(6) $$Y=f\left(x_{1},x_{2},x_{3},\ldots ,x_{k}+e\right)$$

where Y is the response variables for the experiments and ($x_{1},x_{2},x_{3},\ldots ,x_{k}$) are the input variables, *e* is an error, and f is a function that consists of cross-products of the terms that form the polynomial. To optimize the response Y, it is necessary to find an approximation functional relationship between the inputs and the response surface. A polynomial is the functional relationship used by RSM ((Equation (7)) in this kind of work.

(7) $$Y=b_{0}+\sum_{i=1}^{n}b_{i}\cdot X_{i}+\sum_{i=1}^{n}b_{\mathrm{ii}}\cdot X_{i}^{2}+\sum_{i=1}^{n-1}\sum_{j=i+1}^{n}b_{\mathrm{ij}}\cdot X_{i}\cdot X_{j}+e$$

where the first summation is the linear part, the second is the quadratic part and the third is the product of pairs of all inputs of the polynomial. The values of the coefficients b_0_, b_i_, b_ii_, and b_ij_ are calculated by use of a regression analysis to determine the relationship between inputs and outputs. Also, the terms that are selected to form the equation are chosen according to their levels of significance [107]. One computes this level by the analysis of variance (ANOVA) and selecting terms according to the *p*-value obtained. When a problem has more than one output, as does this work, the optimization problem can be solved by using MRS [108]. In this regard, Harrington developed the desirability functions to obtain a compromise between the different outputs (Equations (8) and (9)) and the overall desirability. The latter is defined as the geometric mean of the desirability of each output (Equation (10)), [109].

(8) $$d_{r}^{\max}=\{\begin{array}{cc} 0 & {\mathrm{if}\;f}_{r}\left(X\right)<A\\ {\left(\frac{f_{r}\left(X\right)-A}{B-A}\right)}^{S} & \mathrm{if}\;A\leq f_{r}\left(X\right)\leq B\\ 1 & {\mathrm{if}\;f}_{r}\left(X\right)>B \end{array}$$

(9) $$d_{r}^{\min}=\{\begin{array}{cc} 1 & {\mathrm{if}\;f}_{r}\left(X\right)<A\\ {\left(\frac{f_{r}\left(X\right)-B}{A-B}\right)}^{S} & \mathrm{if}\;A\leq f_{r}\left(X\right)\leq B\\ 0 & {\mathrm{if}\;f}_{r}\left(X\right)>B \end{array}$$

(10) $$D={\left(\prod_{r=1}^{R}d_{r}\right)}^{1/R}$$

In these equations, parameters A and B correspond to the limits of the input ranges, S is an exponent that determines the importance of reaching the target value, X corresponds to the input vector, and f_r_ is the polynomial function that is used to predict the response. A second polynomial degree should be used to optimize one or several responses [108]. The desirability approach involves transforming each estimated response into a unitless utility that is bounded by 0 < d_r_ < 1, where a higher value of d_r_ indicates that the response value is more desirable. The optimization, which was developed with R statistical package, searches for a combination of importance factors that simultaneously satisfy the optimization criteria of each response and input [110].

### 4.2. Combining FEM and MRS to Optimize Mechanical Problems

Over the years, many researchers have used the FEM as an alternative to reduce costs during the design and optimization phases of mechanical problems. The FEM is widely used in industry to analyze engineering problems that are too complicated to solve by classical analytical methods. The object of its application is to reduce the expense incurred by experimental tests. FEM has the disadvantage of requiring a high computational cost especially when the process of fitting the proposed FE models is based only on the experience of the designer through simulations and trial and error. This adjustment process is greatly amplified when the FE model tries to solve non-linear problems, such as mechanical contacts, large deformations and hyper-elastic material behavior. However, the computational cost is reduced with the RSM, which provides an approximation of the same problem that is modeled with FEM [111]. Building regression models based on RSM is a good strategy to reduce the number of simulations that are necessary to model and optimize FE models. These models learn from the most characteristic samples that are obtained from FEM simulations and use their outputs. In this regard, the combination of RSM and FEM has been used widely to optimize many industrial processes. For example, Lin et al. [112] proposed a functionally graded material (FGM) as a potential upgrade to some conventional implant materials like titanium in prosthetic dentistry. In that work, computational bone remodeling and design optimization are used. Based on the results of remodeling, RSM has been adopted to develop a multi-objective optimal design for FGM implantation. Similarly, Sadollah and Bahreininejad [113] investigated the development of an optimal design of an FGM dental implant to promote long-term success. Also, Rungsiyakull et al. [114] combined RSM and FEM to establish a relationship between the surface morphology induced micromechanics and bone remodeling responses to a solid bead coated porous implant. In this case, the RSM was used to relate the major implant coating parameters to the bone responses. More recently, Bahraminasab et al. [115] studied the use of a functionally graded material (FGM) for the femoral component of knee implants. This is attractive because the properties can be designed to vary in a certain pattern to meet the desired requirements at different regions of the knee joint system, thereby decreasing the loosening problem. Therefore, a multi-objective design optimization of a FGM femoral component is conducted in this study by use of the FEM and RSM. The results of using an optimized FGM are then compared to those of using a standard Co–Cr alloy in a femoral component knee implant to demonstrate relative performance. In the current paper, the RSM used the results of FE analysis to search for the optimal parameter that defines correctly the behavior of healthy human lumbar intervertebral disc (IVD) FE models. Eleven parameters with which to define the parameterized FE models were selected. They were C0, C10, Fiber12, Fiber34, Fiber56, Fiber78, Fiber910, Annulus_E, Annulus_μ, Cartil_E, and Cartil_μ. For each of the standard tests, regression models were generated to model the six stiffness and nine bulges of the healthy IVD models when the parameters of the FE models were changed. The optimal combination of the eleven parameters was achieved by applying MRS based on desirability functions according to three different adjustment criteria.

## 5. Results and Discussion

### 5.1. FE Models’ Results

After the parameterized healthy IVD FE models were created, an automatic procedure conducted the 128 simulations according to the design matrix that appears in Table 9. Table 10 shows the stiffness and bulges that were obtained from the FE simulations. They are named as follows and grouped according to the corresponding standard test. For the compression test, the anterior, posterior and lateral bulges values (Comp_bulgeA, Comp_bulgeL, Comp_bulgeP), as well as the stiffness (Comp_stiff), were obtained. The same applies to Shear, Extension, Lateral Bending, Flexion and Torsion tests: Shear_stiff, Exte_bulgeL, Exte_bulgeP, Exte_stiff, LBend_bulgeL, LBend_bulgeP, LBend_stiff, Flex_bulgeL, Flex_bulgeP, Flex_stiff, and Tors_stiff. These 128 values formed the training dataset and were used to generate the regression models. The following subsections show the process to generate and optimize these models.

### 5.2. Analysis of Variance

Equation (6) was fitted with the data that appear in Table 9 and Table 10 to obtain the regression equations for all responses by the use of the RMS “R” package [116]. Each of the responses or outputs from Equations (6) through (20) is shown. They were obtained by a combination of polynomials that are formed by input variables. The responses are: Comp_bulgeA, Comp_bulgeL, Comp_bulgeP, Comp_stiff, Shear_stiff, Exte_bulgeL, Exte_bulgeP, Exte_stiff, LBend_bulgeL, LBend_bulgeP, LBend_stiff, Flex_bulgeL, Flex_bulgeP, Flex_stiff, and Tors_stiff.

(11) $$\begin{array}{l} \mathrm{Comp}\_\mathrm{bulgeA}=0.453125-0.150922\times C10-0.154598\times \;C0-4.4\times 10^{-0.5}\\ \times \mathrm{Fiber}12-2.8\times 10^{-0.5}\times \mathrm{Fiber}56-2.3\times 10^{-0.5}\times \mathrm{Fiber}78-2.5\times 10^{-0.5}\\ \times \mathrm{Fiber}910-0.034134\times \mathrm{Annulus}\_E+0.262148\times \mathrm{Annulus}\_\mu \\ -0.001137\times \mathrm{Cartil}\_E+0.040998\times \mathrm{Cartil}\_\mu \end{array}$$

(12) $$\begin{array}{l} \mathrm{Comp}\_\mathrm{bulge}\;L=0.169454-0.084948\times C10-0.087169\times C0-5\times 10^{-0.5}\\ \times \mathrm{Fiber}12-0.006264\times \mathrm{Annulus}\_E+0.088348\times \mathrm{Annulus}\_\mu \\ -0.001015\times \mathrm{Cartil}\_E+0.031074\times \mathrm{Cartil}\_\mu \end{array}$$

(13) $$\begin{array}{l} \mathrm{Comp}\_\mathrm{bulge}\;P=1.206599-0.91178\times C10-0.915255\times C0\\ -0.113434\times \mathrm{Annulus}\_E+0.442496\times \mathrm{Annulus}\_\mu -0.003303\\ \times \mathrm{Cartil}\_E+0.117189\times \mathrm{Cartil}\_\mu \end{array}$$

(14) $$\begin{array}{l} \mathrm{Comp}\_\mathrm{stiff}=-635.243327+822.048324\times C10+834.364625\times C0\\ +180.06106\times \mathrm{Annulus}\_E+2051.947822\times \mathrm{Annulus}\_\mu \\ +5.673156\times \mathrm{Cartil}\_E \end{array}$$

(15) $$\begin{array}{l} \mathrm{Cort}\_\mathrm{stiff}=-70.312808+199.672441\times C10+199.736145\times C0\\ +0.02166\times \mathrm{Fiber}12+0.055155\times \mathrm{Fiber}34+0.031639\times \mathrm{Fiber}56\\ +0.057449\times \mathrm{Fiber}78+0.044255\times \mathrm{Fiber}910+58.151336\times \mathrm{Annulus}\_E\\ -72.981833\times \mathrm{Annulus}\_\mu +0.926751\times \mathrm{Cartil}\_E-16.717294\times \mathrm{Cartil}\_\mu \end{array}$$

(16) $$\begin{array}{l} \mathrm{Exte}\_\mathrm{bulgeL}=0.083398+0.00016\times \mathrm{Fiber}\_910+0.028063\times \mathrm{Annulus}\_\mu \\ -0.001135\times \mathrm{Cartil}\_E \end{array}$$

(17) $$\begin{array}{l} \mathrm{Exte}\_\mathrm{bulgeP}=-0.243941+0.001987\times \mathrm{Fiber}910+0.76198\times \mathrm{Annulus}\_\mu \\ -0.003522\times \mathrm{Cartil}\_E \end{array}$$

(18) $$\begin{array}{l} \mathrm{Exte}\_\mathrm{stiff}=6.205107-0.01339\times \mathrm{Fiber}910+2.595841\times \mathrm{Annulus}\_\mu \\ +0.008305\times \mathrm{Cartil}\_E \end{array}$$

(19) $$\begin{array}{l} \mathrm{LBend}\_\mathrm{bulgeL}=1.574761-1.014308\times C10-0.929876\times \mathrm{Annulus}\_\mu \\ -0.017706\times \mathrm{Cartil}\_E+1.620387\times \mathrm{Cartil}\_\mu \end{array}$$

(20) $$\begin{array}{l} \mathrm{LBend}\_\mathrm{bulgeP}=2.995354-3.019289\times C10-3.118902\times C0\\ -0.212027\times \mathrm{Annulus}\_E+1.351171\times \mathrm{Annulus}\_\mu -0.023247\times \mathrm{Cartil}\_E\\ +0.84824\times \mathrm{Cartil}\_\mu \end{array}$$

(21) $$\begin{array}{l} \mathrm{LBend}\_\mathrm{stiff}=-1.428516+1.708987\times C10+0.297374\times \mathrm{Annulus}\_E\\ +3.014858\times \mathrm{Annulus}\_\mu +0.041594\times \mathrm{Cartil}\_E-1.896308\times \mathrm{Cartil}\_\mu \end{array}$$

(22) $$\begin{array}{l} \mathrm{Flex}\_\mathrm{bulgeL}=0.125918-0.064104\times C10-0.060597\times C0\\ -3.1e-0.5\times \mathrm{Fiber}12-0.00418\times \mathrm{Annulus}\_E+0.04945\times \mathrm{Annulus}\_\mu \\ -0.000311\times \mathrm{Cartil}\_E-0.019064\times \mathrm{Cartil}\_\mu \end{array}$$

(23) $$\begin{array}{l} \mathrm{Flex}\_\mathrm{bulgeP}=0.681909-0.920479\times C10-0.921496\times C0\\ -0.048626\times \mathrm{Annulus}\_E-0.113105\times \mathrm{Annulus}\_\mu +0.039252\times \mathrm{Cartil}\_\mu \end{array}$$

(24) $$\begin{array}{l} \mathrm{Flex}\_\mathrm{stiff}=-0.697824+1.433372\times C10+1.443693\times C0\\ +0.269714\times \mathrm{Annulus}\_E+2.099688\times \mathrm{Annulus}\_\mu +0.013196\times \mathrm{Cartil}\_E\\ -0.368862\times \mathrm{Cartil}\_\mu \end{array}$$

(25) $$\begin{array}{l} \mathrm{Tors}\_\mathrm{stiff}=-2.079685+1.056596\times C10+0.901329\times C0\\ +0.00263\times \mathrm{Fiber}34-0.000469\times \mathrm{Fiber}56+0.001202\times \mathrm{Fiber}78\\ +0.908448\times \mathrm{Annulus}\_E-0.905043\times \mathrm{Annulus}\_\mu +0.013001\times \mathrm{Cartil}\_E\\ -0.521362\times \mathrm{Cartil}\_\mu \end{array}$$

In order determine whether the variables that were used in the linear regression models are statistically significant, an ANOVA test was developed. Table 11, Table 12, Table 13, Table 14, Table 15, Table 16, Table 17, Table 18, Table 19, Table 20, Table 21, Table 22, Table 23, Table 24 and Table 25 show the ANOVA results. The tables show that most of the variables have a *p*-value of less than 0.01. This means that the models are statistically significant. In addition, the multiple correlation coefficient (R^2^) was calculated as a measure of the variation around the mean that the regression model produced. The results show that all values of R^2^ are close to one. This indicates that these models possess good predictive capacity.

Also, MAE and RMSE are calculated to determine the generalization capacity of the regression models that were obtained by using the results of the design matrix (inputs) from Table 9 and their corresponding outputs from Table 10 according to Equations (26) and (27).

(26) $$MAE=\frac{1}{m}\cdot \sum_{k=1}^{m}\left|Y_{k\;FEM}-Y_{k\;\mathrm{Model}}\right|$$

(27) $$RMSE=\sqrt{\frac{1}{m}\cdot \sum_{k=1}^{m}{\left(Y_{k\;FEM}-Y_{k\;\mathrm{Model}}\right)}^{2}}$$

Table 26 shows the prediction errors, when the maximum error corresponds to Exte_stiff (MAE equal to 13.97% and RMSE equal to 21.81%). and the minimum error corresponds to Shear_stiff (MAE equal to 1.74% and RMSE equal to 2.19%).

**Figure 7:**
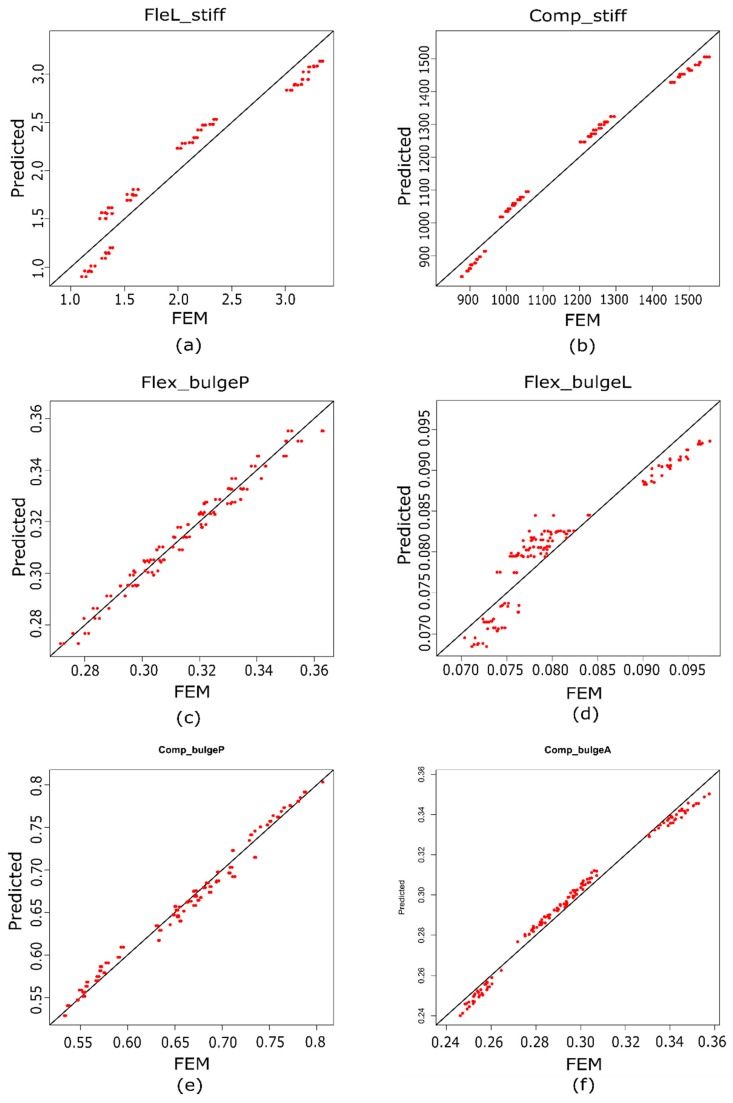
Scatter diagram of (**a**) lateral bending stiffness; (**b**) compression stiffness; (**c**) flexion bulge posterior; (**d**) flexion bulge lateral; (**e**) compression bulge posterior; and (**f**) compression bulge anterior.

Figure 7 shows the relationship between some of the actual values that were obtained experimentally with FEM (Table 10) and the predicted (regression models) values of LBend_stiff (Figure 7a), Comp_stiff (Figure 7b), Flex_bulgeP (Figure 7c), Flex_bulgeL (Figure 7d), Comp_bulgeP (Figure 7e), and Comp_bulgeA (Figure 7f). These figures indicate that the correlations between the real values and the predicted ones are high, and the residuals that were obtained were small.

Additionally, 30 new FE models were created to test the proposed regression models with parameters that had not been used previously to generate regression models. Table 27 shows the errors incurred during the testing stage, when the maximum error corresponds to Tor_stiff (MAE equal to 10.06% and RMSE equal to 19.50%) and the minimum error corresponds to Flex_bulgeP (MAE equal to 2.86% and RMSE equal to 3.19%). The errors show that the adjustment between the regression models and the results obtained from the FE models is almost accurate. This also demonstrates its good capacity for generalization.

**Figure 8:**
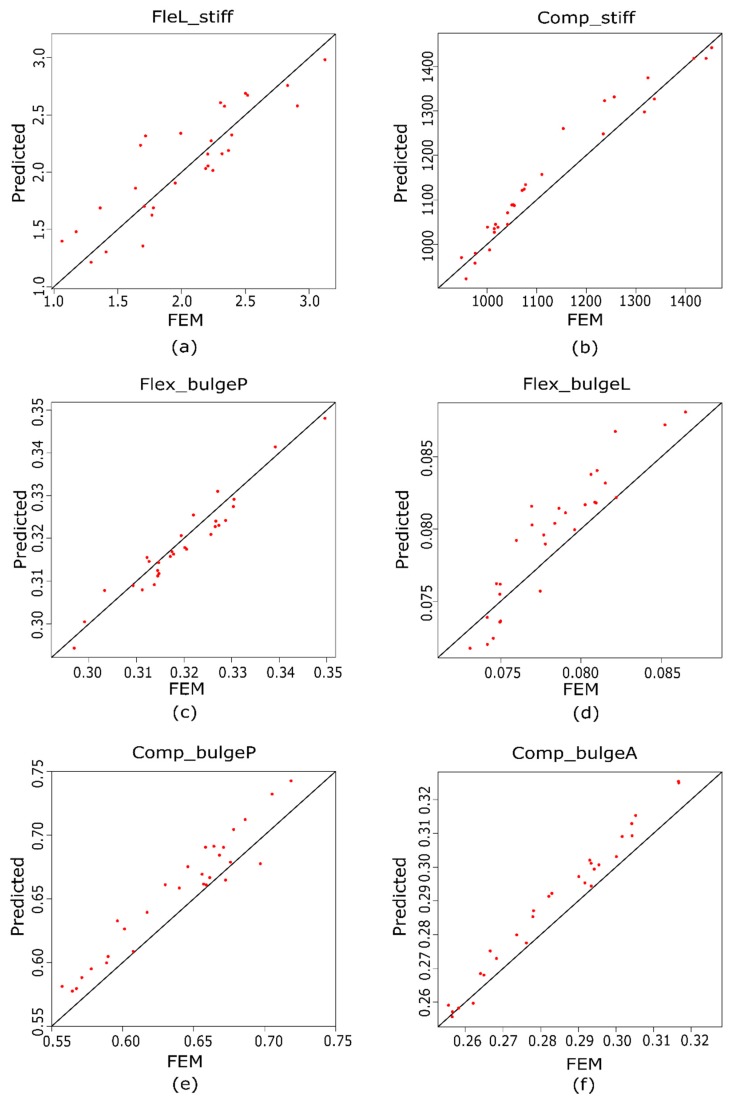
Test scatter diagram of (**a**) lateral bending stiffness; (**b**) compression stiffness; (**c**) flexion bulge posterior; (**d**) flexion bulge lateral; (**e**) compression bulge posterior, and (**f**) compression bulge anterior.

Figure 8 shows the relationship between some of the 30 additional FE models that were implemented to test the regression models and their corresponding FE simulations. Figure 7 shows the values of LBend_stiff (Figure 8a), Comp_stiff (Figure 8b), Flex_bulgeP (Figure 8c), Flex_bulgeL (Figure 8d), Comp_bulgeP (Figure 8e), and Comp_bulgeA (Figure 8f). The figures indicate that the correlations are high, whereas the residuals that were obtained are small, which indicate that these regression models are adequate for the prediction of IVD behavior.

### 5.3. Multiple Response Optimization

Table 28, Table 29 and Table 30 show a combination of the eleven material parameter (inputs) that were studied in searching for the optimal behavior of human intervertebral lumbar disc FE models when using the desirability functions with the “R” package [110]. Three different adjustment criteria were considered. The first column of the tables shows the material parameters (inputs) and stiffness values and bulges (outputs) that were studied. The second column shows the goals that were established in the goal setting process for both inputs and outputs when different adjustment criteria are considered. The third column shows the optimal values to define the behavior of the FE model and the last column shows the desirability values. Table 28 shows the results when all material parameters, as well as stiffness and bulges, were considered to have the same level of importance (equal to “inRange”). In this case, the value of the overall desirability was 0.625. Also, the table shows that some of the values that were obtained are very close to the targets that were proposed. For example, the target proposed for the Exte_bulgeL was 0.1 and the optimal value that was obtained was 0.100 with a desirability value of 1. In contrast, the proposed target for the Comp_bulgeL was 0.35 and the optimal value that was obtained was 0.0970 with a desirability value of 0.244.

Table 29 shows the results when the stiffness was considered to have a higher level of importance than bulges. This table shows that the material parameters that were obtained for all different goals required in the first criteria are very similar, although the value of overall desirability was 0.817, which is higher than the first criteria. Also, the target for the parameters of Shear_stiff and Tors_stiff were 300 and 2.1, and the values obtained were 300.000 (desirability = 1) and 3.456 (desirability = 0.428), respectively.

Table 30 shows the results that were obtained with the third criterion when the bulges were considered to be the most important. Analogously to the other adjustment criteria that were previously studied, the material parameters that have been obtained are very similar for all different goals. In this case, the value of overall desirability was 0.554, which is the lowest result of the three criteria that are studied in this paper. Also, it is observed that the targets for the parameters of Exte_bulgeL and Comp_bulgeL were 0.1 and 0.35, and the values obtained were 0.103 (desirability = 0.947) and 0.101 (desirability = 0.257), respectively.

Finally, three new FE models were simulated with the eleven different optimal material parameters that considered the three different adjustment criteria. These FE models were simulated again by the same standard test (Compression, Flexion, etc.) in order to compare the methodology proposed for adjust the optimal parameters. Table 31 shows a comparison of the results with the FE models using the optimized parameters, the optimal results from the regression models using the RMS with desirability functions and the experimental standard test. In order to compare the different errors that were obtained using three different adjustment criteria that were studied in this case, different MAE were obtained from the normalized data. The data is commonly normalized in statistical processes to transform all variables to the same scale (from 0–1). The transformation was achieved in this case by subtracting the minimum value from each original value and dividing the result by the range of each variable according to Equation (28).

(28) $$Y_{k,norm}=\frac{Y_{k}-\min\left(Y\right)}{\max\left(Y\right)}$$

where *Y_k,norm_* were the normalized outputs that were obtained from the results of the FE models with the optimized parameters and the outputs that were obtained experimentally from the standard test. The first column of the table shows the stiffness and bulges (outputs) that were studied. The second, third, and fourth columns show, respectively, the results obtained from the FE models with the optimized parameters for each of the different criteria that were considered. The fifth column shows the values of the standard test, and the last column shows the normalized MAE that was obtained. It can be seen in the table that the normalized MAE of the three criteria considered are very similar (Criteria 1 = 0.2782, Criteria 2 = 0.2795 and Criteria 3 = 0.2788). In contrast, the normalized MAE obtained for each of the outputs is lower when predicting the Shear_stiff (MAE = 0.01) and greater when predicting Comp_bulgeL (MAE = 0.73). The reason for this difference may be that the proposed FE model is generally less accurate in predicting bulges than stiffness. In addition, all of the values of MAE that were obtained for each of the different outputs or stiffness and bulges are in acceptable agreement.

## 6. Conclusions

This paper sets out a fully automated method that combines the finite element method (FEM) and multi-response surface method (MRS) based on desirability functions. This work looks for the optimal parameters that will correctly define the behavior of a medium-sized healthy human lumbar intervertebral disc (IVD) models based on FEM. First, based on standard tests (compression, flexion, extension, shear, lateral bending, and torsion), three-dimensional parameterized finite element (FE) models were generated. Then, 11 parameters were selected to define the parameterized intervertebral lumbar disc FE models. For each of the standard tests, regression models were generated for modeling the six stiffness and nine bulges of the healthy IVD models when the parameters of the FE models were varied. The optimal combination of the 11 parameters was achieved by applying MRS based on desirability functions according to three different adjustment criteria. The first criterion considered all of the material parameters (inputs), as well as the stiffness and bulges (outputs), with the same level of importance (equal to “inRange”). The second criterion considered, with a higher level of importance the stiffness, then bulges (goal of stiffness = target), whereas the third criteria considered, the bulges with a higher level of importance, then stiffness (goal of bulges = target). The best fit of the FE model parameters was achieved with the proposed second criterion. This produced a value for the normalized MAE of 0.2795. However, the results were very similar for the first and the third criteria that were considered. These were, respectively, MAE = 0.2782 and MAE = 0.2788. In contrast, the normalized MAE obtained for each of the outputs was lower when predicting the Shear_stiff (MAE = 0.01) and greater when predicting Comp_bulgeL (MAE = 0.73). This reason for this difference may be that the proposed FE model is generally less accurate in predicting bulges than stiffness. The MAE obtained from each criterion that was studied demonstrated that the proposed method is a powerful tool for adjusting healthy IVD FE models when there are many parameter and the stiffness and number of bulges to which the models must be adjusted, are high.

## Figures and Tables

**Table 1:** Range of the material parameters proposed to define the behavior of human intervertebral lumbar disc models based on the finite element method (FEM).

	Nucleus Pulposus				Cartilage Endplate		Annulus Ground				Annulus Fibers					
Authors	FE Parameters		FE Parameters		FE Parameters		FE Parameters				FE Parameters					
	Mooney-Rivlin Max.		Isotropic		Isotropic		Mooney-Rivlin		Isotropic		F1	F2	F3	F4	F5	
	C10	C0	E	Μ	E	μ	C10	C0	E	μ	E					μ
Kim and Chun (2015) [36]	-	-	1	0.4999	24	0.40	-	-	4.2	0.45	550–358					0.3
Dicko et al. (2015) [37]	-	-	1	0.4999	24	0.40	0.18	0.045	-	0.45	Non-linear stress-strain curve					
González et al. (2015) [42]	0.12	0.03	0.5 < E < 1	0.4 < μ < 0.5	20	0.3	-	-	0.75–5	0.35–0.5	-					-
Ibarz, Elena et al. (2014) [47]	0.0343	0.1369	-	-	-	-	-	-	4.2	0.45	550	503	455	408	360	0.3
Tsouknidas et al (2012) [35]	-	-	0.2	0.4999	-	-	-	-	4.2	0.45	550	485	440	420	360	0.45
Ayturk, U.M. (2010) [38]	-	-	1	0.4999	23.8	0.8	C10 = 0.0146; C20 = −0.0189; C30 = 0.041				a3 = 0.03; b3 = 120					
Schmidt, Kettler (2007) [44]	0.12	0.03	-	0.4999	23.8	0.8	0.10	0.05		0.45	* Stress-strain curve by Shirazi: σ = 23,000 × ε^1.9^					
Rohlmann et al. (2006) [46]	0.10	0.09	-	0.4999	23.8	0.8	0.348	0.3	0.42	0.45	* Stress-strain curve by Shirazi: σ = 23,000 × ε^1.9^					
Rohlmann, Zander (2006) [45]	0.10	0.09	-	0.4999	-	-	0.348	0.3	0.42	0.45	* Stress-strain curve by Shirazi: σ = 23,000 × ε^1.9^					
Grauer et al. (2006) [39]	-	-	1	0.4999	-	-	-	-	4.2	0.45	175	175	175	175	175	-
Dietrich, M. et al. (2005) [51]	-	-	0.012	0.4999	-	-	-	-	10	0.35	-	-	-	-	-	-
Denoziére, G. et al (2004) [2]	-	-	0.1	0.4999	12	0.3	-	-	4.2	0.45	550	485	440	420	360	0.3
Baroud et al. (2003) [30]	0.12	0.03	-	-	-	-	-	-	8	0.45	500	485	420	360	-	-
Pitzen et al. (2002) [33]	-	-	0.1	0.4999	-	-	-	-	4.2	0.45	500	485	420	360	-	-
Dooris et al. (2001) [40]	-	-	1	0.49	-	-	-	-	-	-	-	-	-	-	-	-
Eberlain et al. (2001) [52]	Incompress. Fluid		-	-	23.8	0.4	0.348	0.3	4	0.4	* Stress-strain curve by Shirazi:σ = 23,000 × ε^1.9^					
Martínez et al. (1997) [49]	-	-	-	-	20	0.3	-	0.3	-	-	-	-	-	-	-	-
Lu et al. (1996) [48]	-	-	-	-	20	0.3	-	-	4.2	0.45	-	-	-	-	-	-
Smit et al. (1997) [41]	0.12	0.09	0.5 < E < 1	0.4999	-	-	-	-	-	-	-	-	-	-	-	-
Sharma et al. (1995) [34]			0.1	0.4999	-	-	-	-	4.2	0.5						
Lavaste et al. (1992) [43]	-	-	1 < E < 4	0.5	-	-	-	-	-	-	-	-	-	-	-	-
Shirazi-Adl et al. (1984) [11]	Incompress. Fluid		-	-	-	-	-	-	4.2	0.45	σ = 23,000 × ε^1.9^					

Yeoh material. Material coefficients: C10 = 0.0146, C20 = −0.0189, C30 = 0.041; a3 = 0.03, b3 = 120.0 (b3 is unitless). C10 = 0.0343 MPa; C0 = 0.1369 MPa. An elastic analysis with a Young modulus of 1.0 MPa and Poisson ratio of 0.49 was conducted with similar results and a volume change of less than 0. * is Stress-strain curve by Shirazi et al. [11]: in this case, ε is the value for the deformation and σ is the stress.

**Table 2:** Summary of stiffness, bulges, and compression loads from various authors.

**Compression Test**			
**Authors**	**Stiffness (N/mm)**	**Load (N)**	**Range of Load (N)**
Moroney et al. (1988) [68]	500	74	
Brown et al. (2002) [69]	400	200	<400
Keller et al. (1987) [70]	247	253	
Berkson et al. (1979) [8]	800	400	
Nachemson et al. (1979) [67]	571	500	
Rostedt et al. (1998) [5]	810	500	
Stokes et al. (2002) [65]	510	500	850–500
Panjabi et al. (1984) [66]	750	600	
Gardner-Morse et al. (2004) [7]	2420	850	
Hirsh and Nachemson (1954) [54]	700	1000	
Schultz et al. (1973) [61]	1500	1000	
González Gutierrez (2012) [3]	833	1000	
González Gutierrez (2012) [3]	933	1000	
González Gutierrez (2012) [3]	1089	1000	5500–1000
Markolf (1970) [62]	1800	1800	
Virgin (1951) [60]	2500	4500	
Rolander and Blair (1975) [63]	3000	5000	
Brown et al. (1957) [64]	2300	5300	
	**Bulges Values**		
**Authors**	**Anterior/Post/Lateral (mm)**	**Load (N)**	
Reuber et al. (1982) [58]	-/0.24/0.66	400	
Schmidt, Kettler (2007) [44]	0.7 to 0.9	500	
Shirazi-Adl et al. (1984) [11]	0.5/0.75/0.35	500	
Shirazi-Adl et al. (1984) [11]	0.7/1/0.4	720	
Reuber et al. (1982) [58]	-/0.34/0.8	800	
Brinckmann et al. (1991) [59]	0.15	1000	
González Gutierrez (2012) [3]	0.69	1000	
Shirazi-Adl et al. (1984) [11]	0.8/1.5/0.6	1000	
Nachemson, A. (1960) [71]	-	2000	
Denozière (2004) [2]	0.5/0.7/0.4	2500	
Klein et al. (1983) [57]	0.6	-	

**Table 3:** Stiffness, bulge values, and flexion-extension loads from various authors.

**Flexion/Extension Test**		
**Authors**	**Stiffness Values (Nm/°)**	**Load (Nm)**
Guan et al. (2007) [75]	0.82/1.53	4
Busscher et al. (2009) [76]	0.8	4
Busscher et al. (2010) [77]	0.8	5
González Gutierrez (2012) [3]	1.18/1.38	5
Patwardhan et al. (2003) [78]	1.33	8
White and Panjabi (1978) [79]	0.8/2	10
Nachemson et al. (1979) [67]	2.03/3.53	10
Gardner-Morse et al. (2004) [7]	2.04	10
Schultz et al. (1979) [9]	1.92/3.55	10.6
Adams et al. (1980) [74]	1.34	10.7
Schultz et al. (1973) [61]	4.5	20
Brown et al. (2002) [69]	2	20
Miller et al. (1986) [72]	5.51/7.60	70
Adams et al. (1996) [73]	7.3	80
	**Bulges Values**	
**Authors**	**Anterior/Post/Lateral (mm)**	**Load (Nm)**
Denoziére, G. et al. (2004) [2]	1.3/1.9/2.6	10
Reuber et al. (1982) [58]	-/0.73/0.07	3.9
Reuber et al. (1982) [58]	-/1.11/0.21	7.9

**Table 4:** Stiffness values and lateral bending loads from various authors.

**Lateral Bending Test**		
**Authors**	**Stiffness Values (Nm/°)**	**Load (Nm)**
Guan et al. (2007) [75]	0.76	4
Busscher et al. (2009) [76]	0.5	4
Busscher et al. (2010) [77]	0.6	5
González Gutierrez (2012) [3]	1.58	5
White and Panjabi (1978) [79]	0.9	10
Nachemson et al. (1979) [67]	1.1	10
Gardner-Morse et al. (2004) [7]	1.29	10
Schultz et al. (1979) [9]	2	10.6
Schultz et al. (1973) [61]	2.8	20
Miller et al. (1986) [72]	4.35	60
	**Bulges Values**	
**Authors**	**Anterior/Post/Lateral (mm)**	**Load (Nm)**
Reuber et al. (1982) [58]	-/0.49/0.83	3.9
Reuber et al. (1982) [58]	-/1.13/2.11	9.8

**Table 5:** Stiffness and loads for the shear and torsion tests by various authors.

**Shear Test**		
**Authors**	**Stiffness (N/mm)**	**Load (N)**
Moroney et al. (1988) [68]	60	20
Markolf (1970) [62]	260	150
Miller et al. (1986) [72]	115	150
Liu et al. (1975) [6]	300	450
Weisse et al. (2012) [80]	830	950
Schultz et al. (1979) [9]	1000	980
Schultz et al. (1973) [61]	685	1000
**Torsion Test**		
**Authors**	**Stiffness (Nm/°)**	**Load(Nm)**
Busscher et al. (2009) [76]	2.5	4
Busscher et al. (2010) [77]	1.6	5
González Gutierrez (2012) [3]	4.4	5
Haughton et al. (1999) [83]	7	6.6
Adams et al. (1981) [82]	1.44	7.4
White and Panjabi (1978) [79]	2.22	10
Nachemson et al. (1979) [67]	8.48	10
Gardner-Morse et al. (2004) [7]	2.1	10
Schultz et al. (1979) [9]	7.07	10.6
Schultz et al. (1973) [61]	4.5	30
Farfan et al. (1970) [81]	2	31
Miller et al. (1986) [72]	10.9	70

**Table 6:** Stiffness and bulge values selected from the standard tests that are used to adjust the parameters that define the behavior of the finite element (FE) model of the intervertebral lumbar disc.

**Test**	**Author**	**Load Used**	**Stiffness**		
Compression	Rostedt et al. (1998) [5]	500 N	810 N/mm		
Flexion	González Gutierrez (2012) [3]	5 Nm	1.18 Nm/°		
Extension	Guan et al. (2007) [75]	4 Nm	1.53 Nm/°		
Lateral Bending Bending	Schultz et al. (1979) [9]	10.6 Nm	2.0 Nm/°		
Shear	Liu et al. (1975) [6]	450 N	300 N/mm		
Torsion	Gardner-Morse et al. (2004) [7]	10 Nm	2.1 Nm/°		
**Test**	**Authors**	**Load Used**	**Bulge**	**Bulg**	**Bulge**
			**Anterior (mm)**	**Posterior (mm)**	**Lateral (mm)**
Compression	Shirazi-Adl et al. (1984) [11]	500 N	0.5	0.75	0.35
Flexion	Reuber et al. (1982) [58]	3.9 Nm	-	0.73	0.07
Extension	Reuber et al. (1982) [58]	3.9 Nm	-	0.24	0.1
Lateral Bending	Reuber et al. (1982) [58]	9.8 Nm	-	1.13	2.11

**Table 7:** Range of the proposed material parameters for defining the behavior of the human intervertebral lumbar disc models based on FEM.

Tissue	FE Parameters		Tissue	FE Parameters	
	Min.	Max.		Min.	Max.
**Nucleus Pulposus**			**Annulus Fibrosus**		
C10	0.11	0.14	Fiber12	515.0	550.0
C0	0.02	0.04	Fiber34	503.0	515.0
**Endplate**			Fiber56	455.0	503.0
E	23.0	55.0	Fiber78	408.0	455.0
μ	0.3	0.4	Fiber910	360.0	408.0
-	-	-	E Annulus Fibrosus	4.0	4.2
-	-	-	μ Annulus Fibrosus	0.25	0.45

**Table 8:** Range of the material parameters proposed to define the behavior of human intervertebral lumbar disc models based on FEM.

Summary of Anatomical Dimensions of L1–L5								
Authors	Group Size (n)	Lumbar Level	Sex	Mean Age (From…to…)	Width (mm)	Depth (mm)	Height (mm)	Area (cm^2^)
Rostedt et al. (1998) [5]	4	L3–L4	-	45	-	-	12	-
Schultz et al. (1979) [9]	1	L1–L5	male	35	-	-	-	1590
Schultz et al. (1979) [9]	1	L1–L5	male	40	-	-	-	1680
Schultz et al. (1979) [9]	1	L1–L5	male	53	-	-	-	1500
Zhou et al. (2000) [88]	55	L3–L5	male	50 (22–80)	53	37.5	12.2	1492 ± 173.8
Zhou et al. (2000) [88]	71	L3–L5	female	49 (22–80)	50.5	35.4	11.3	1492 ± 173.8
Panjabi (1992) [90]	60	L1–L5	-	46.3 (19–59)	48.1	34.7	-	-
Eijkelkamp (2002) [91]	60	L1–L5	-	(18–65)	-	-	13.5	-
Nissan and Gilad (1986) [92]	157	L1–L5	-	26.8 (20–38)	-	34.6	10.8	-
Tibrewal and Pearcy (1985) [93]	11	L1–L5	-	29.5 (25–36)	-	33	9.8	-
Wolf et al. (2001) [94]	55	L1–L5	-	(20–90)	44.1	31.7	-	-
Amonoo-Kuofi (1991) [95]	305	L1–L5	male	(10–64)	-	42.8	13.5	-
Amonoo-Kuofi (1991) [95]	310	L1–L5	female	(10–61)	-	39.9	13	-
Schmidt et al. (2006) [24]	-	L4–L5	-	-	58.7	37.4	-	-
Kim and Chun (2015) [36]	1	L4–L5	male	46	-	-	-	1119
González et al. (2015) [42]	5	L2–L3	male/female	(65–75)	-	-	9.9	1739
González et al. (2015) [42]	5	L4–L5	male/female	(65–75)	-	-	10	1951
Shirazi-Adl et al. (1984) [11]	1	L2–L3	female	29	49.2	34	11	1371
Smit et al. (1997) [41]	-	L4	-	-	42	35	-	-
Ibarz, Elena et al. (2014) [47]	25	L5–S1		27.4				
Ayturk, U.M. (2010) [38]	-	L1–L5	female	49				-
Weisse et al. (2012) [80]	-	L4–L5	male	43	50.3	33.7	12.8	-
Denozière (2004) [2]	-	L3–L4	-	-	50	35	10	1440

**Table 9:** Design matrix for the simulation of FE models when considering combination of 128 material parameters (inputs).

Run	C10	C0	Fiber 12	Fiber 34	Fiber 56	Fiber 78	Fiber 910	Annulus E	Annulus μ	Cartil E	Cartil μ
1	0.11	0.02	515	503	455	408	360	4	0.25	55	0.4
2	0.14	0.02	515	503	455	408	360	4.2	0.45	23	0.3
3	0.11	0.04	515	503	455	408	360	4.2	0.45	23	0.4
4	0.14	0.04	515	503	455	408	360	4	0.25	55	0.3
5	0.11	0.02	550	503	455	408	360	4.2	0.45	55	0.3
6	0.14	0.02	550	503	455	408	360	4	0.25	23	0.4
7	0.11	0.04	550	503	455	408	360	4	0.25	23	0.3
8	0.14	0.04	550	503	455	408	360	4.2	0.45	55	0.4
9	0.11	0.02	515	515	455	408	360	4.2	0.25	23	0.4
10	0.14	0.02	515	515	455	408	360	4	0.45	55	0.3
…	…	…	…	…	…	…	…	…	…	…	…
120	0.14	0.04	550	503	503	455	408	4	0.45	23	0.4
121	0.11	0.02	515	515	503	455	408	4	0.25	55	0.4
122	0.14	0.02	515	515	503	455	408	4.2	0.45	23	0.3
123	0.11	0.04	515	515	503	455	408	4.2	0.45	23	0.4
124	0.14	0.04	515	515	503	455	408	4	0.25	55	0.3
125	0.11	0.02	550	515	503	455	408	4.2	0.45	55	0.3
126	0.14	0.02	550	515	503	455	408	4	0.25	23	0.4
127	0.11	0.04	550	515	503	455	408	4	0.25	23	0.3
128	0.14	0.04	550	515	503	455	408	4.2	0.45	55	0.4

**Table 10:** Results of the simulation of FE models when a combination of 128 material parameters are considered in Table 9.

Run	Comp BulgeA	Comp BulgeL	Comp BulgeP	Comp Stiff	Shear Stiff	Exte BulgeL	Exte BulgeP	Exte Stiff	LBend BulgeL	LBend BulgeP	LBend Stiff	Flex BulgeL	Flex BulgeP	Flex Stiff	Tors Stiff
1	0.265	0.089	0.593	984.510	305.752	0.095	0.574	1.744	0.773	1.085	1.998	0.076	0.351	1.635	3.593
2	0.337	0.133	0.730	1271.883	281.371	0.137	0.830	2.121	0.970	1.929	1.581	0.095	0.288	1.762	3.264
3	0.347	0.140	0.758	1253.827	275.927	0.139	0.861	2.094	1.386	2.136	1.320	0.093	0.301	1.646	3.150
4	0.256	0.085	0.553	1017.076	315.425	0.096	0.566	1.779	0.798	1.051	2.257	0.073	0.303	1.686	3.640
5	0.298	0.094	0.665	1500.886	306.597	0.096	0.696	2.406	0.617	1.254	3.268	0.080	0.321	2.227	3.618
6	0.290	0.111	0.694	897.694	284.611	0.135	0.667	1.627	1.520	1.805	1.160	0.076	0.331	1.348	3.191
7	0.286	0.108	0.683	891.309	285.801	0.135	0.644	1.619	1.349	1.653	1.322	0.082	0.334	1.394	3.271
8	0.295	0.092	0.634	1548.473	316.704	0.095	0.685	2.461	0.672	1.188	3.215	0.076	0.280	2.309	3.682
9	0.290	0.113	0.711	901.414	289.552	0.132	0.661	1.661	1.531	1.892	1.130	0.078	0.349	1.338	3.320
10	0.304	0.095	0.670	1474.443	300.617	0.099	0.728	2.351	0.625	1.237	3.229	0.081	0.301	2.192	3.447
…	…	…	…	…	…	…	…	…	…	…	…	…	…	…	…
120	0.345	0.136	0.752	1256.486	278.278	0.139	0.875	2.063	1.322	2.070	1.382	0.090	0.284	1.673	3.016
121	0.260	0.088	0.596	989.416	313.213	0.094	0.574	1.749	0.778	1.086	1.990	0.076	0.352	1.635	3.665
122	0.333	0.131	0.731	1276.741	287.840	0.135	0.829	2.127	0.961	1.928	1.583	0.094	0.289	1.766	3.318
123	0.342	0.139	0.760	1258.926	282.358	0.137	0.860	2.100	1.374	2.137	1.325	0.092	0.302	1.652	3.201
124	0.252	0.085	0.555	1022.062	322.997	0.095	0.565	1.783	0.801	1.052	2.250	0.073	0.304	1.686	3.723
125	0.294	0.092	0.667	1507.286	314.067	0.094	0.693	2.414	0.614	1.251	3.262	0.079	0.322	2.230	3.670
126	0.286	0.110	0.697	901.330	291.307	0.133	0.667	1.630	1.524	1.809	1.160	0.076	0.332	1.350	3.255
127	0.282	0.107	0.685	894.800	292.534	0.134	0.644	1.621	1.351	1.654	1.321	0.082	0.335	1.395	3.333
128	0.291	0.091	0.635	1555.367	324.328	0.093	0.684	2.469	0.670	1.186	3.210	0.076	0.281	2.310	3.740

**Table 11:** Analysis of variance (ANOVA) table for a compression bulge anterior linear model.

Compression Bulge Anterior BulgeL						
Var.	Df	Sum of Sq.	Mean Square	F Value	*p*-Value	Significance Code
C10	1	0.00066	0.00066	35.8	2.41 × 10^−8^	***
C0	1	0.00031	0.00031	16.7	8.01 × 10^−5^	***
Fiber12	1	0.00008	0.00008	4.1	4.47 × 10^−2^	*
Fiber56	1	0.00006	0.00006	3.1	7.96 × 10^−2^	.
Fiber78	1	0.00004	0.00004	2.0	1.58 × 10^−1^	
Fiber910	1	0.00005	0.00005	2.6	1.09 × 10^−1^	
Annulus_E	1	0.00149	0.00149	81.5	4.25 × 10^−15^	***
Annulus_μ	1	0.08796	0.08796	4805.3	<2.2 × 10^−16^	***
Cartil_E	1	0.04237	0.04237	2314.5	<2.2 × 10^−16^	***
Cartil_μ	1	0.00054	0.00054	29.4	3.24 × 10^−7^	***
Residuals	117	0.00214	0.00002			

Significance codes: 0 ‘***’ 0.001 ‘**’ 0.01 ‘*’ 0.05 ‘.’ 0.1 ‘ ’ 1.

**Table 12:** ANOVA table for a compression bulge lateral linear model.

Compression Bulge Lateral						
Var.	Df	Sum of Sq.	Mean Square	F Value	*p*-Value	Significance Code
C10	1	0.00021	0.00021	8.3	4.73 × 10^−3^	**
C0	1	0.00010	0.00010	3.9	5.12 × 10^−2^	.
Fiber12	1	0.00010	0.00010	4.0	4.89 × 10^−2^	*
Annulus_E	1	0.00005	0.00005	2.0	1.60 × 10^−1^	
Annulus_μ	1	0.00999	0.00999	398.4	<2.2 × 10^−16^	***
Cartil_E	1	0.03378	0.03378	1347.0	<2.2 × 10^−16^	***
Cartil_μ	1	0.00031	0.00031	12.3	6.31 × 10^−4^	***
Residuals	1200	0.00301	0.00003			

Significance codes: 0 ‘***’ 0.001 ‘**’ 0.01 ‘*’ 0.05 ‘.’ 0.1 ‘ ’ 1.

**Table 13:** ANOVA table for a compression bulge posterior linear model.

Compression Bulge Posterior						
Var.	Df	Sum of Sq.	Mean Square	F Value	*p*-Value	Significance Code
C10	1	0.02444	0.02444	323.4	<2.2 × 10^−16^	***
C0	1	0.01072	0.01072	141.9	<2.2 × 10^−16^	***
Annulus_E	1	0.01647	0.01647	217.9	<2.2 × 10^−16^	***
Annulus_μ	1	0.25063	0.25063	3316.3	<2.2 × 10^−16^	***
Cartil_E	1	0.35753	0.35753	4730.9	<2.2 × 10^−16^	***
Cartil_μ	1	0.00439	0.00439	58.2	6.11 × 10^−12^	***
Residuals	121	0.00914	0.00008			

Significance codes: 0 ‘***’ 0.001 ‘**’ 0.01 ‘*’ 0.05 ‘.’ 0.1 ‘ ’ 1.

**Table 14:** ANOVA table for a compression stiffness linear model.

Compression Stiffness						
Var.	Df	Sum of Sq.	Mean Square	F Value	*p*-Value	Significance Code
C10	1	19,462	19,462	14.7	2.00 × 10^−4^	***
C0	1	8911	8911	6.7	1.06 × 10^−2^	*
Annulus_E	1	41,500	41,500	31.4	1.34 × 10^−7^	***
Annulus_μ	1	5,389,427	5,389,427	4074.1	<2.2 × 10^−16^	***
Cartil_E	1	1,054,628	1,054,628	797.2	<2.2 × 10^−16^	***
Residuals	122	161,387	1323			

Significance codes: 0 ‘***’ 0.001 ‘**’ 0.01 ‘*’ 0.05 ‘.’ 0.1 ‘ ’ 1.

**Table 15:** ANOVA table for a shear stiffness linear model.

Shear Stiffness						
Var.	Df	Sum of Sq.	Mean Square	F Value	*p*-Value	Significance Code
C10	1	1148.2	1148.2	438.8	<2.2 × 10^−16^	***
C0	1	510.6	510.6	195.1	<2.2 × 10^−16^	***
Fiber12	1	18.4	18.4	7.0	9.15 × 10^−3^	**
Fiber34	1	14.0	14.0	5.4	2.24 × 10^−2^	*
Fiber56	1	73.8	73.8	28.2	5.34 × 10^−7^	***
Fiber78	1	233.3	233.3	89.2	4.82 × 10^−16^	***
Fiber910	1	144.4	144.4	55.2	2.02 × 10^−11^	***
Annulus_E	1	4328.4	4328.4	1654.0	<2.2 × 10^−16^	***
Annulus_μ	1	6817.7	6817.7	2605.3	<2.2 × 10^−16^	***
Cartil_E	1	28,143.4	28,143.4	10,754.5	<2.2 × 10^−16^	***
Cartil_μ	1	89.4	89.4	34.2	4.73 × 10^−8^	***
Residuals	116	303.6	2.6			

Significance codes: 0 ‘***’ 0.001 ‘**’ 0.01 ‘*’ 0.05 ‘.’ 0.1 ‘ ’ 1.

**Table 16:** ANOVA table for an extension bulge lateral linear model.

Extension Bulge Lateral Stiffness						
Var.	Df	Sum of Sq.	Mean Square	F Value	*p*-Value	Significance Code
Fiber910	1	0.00189	0.00189	12.9	4.62 × 10^−4^	***
Annulus_μ	1	0.00101	0.00101	6.9	9.74 × 10^−3^	**
Cartil_E	1	0.04221	0.04221	288.7	<2.2 × 10^−16^	***
Residuals	1244	0.01813	0.00015			

Significance codes: 0 ‘***’ 0.001 ‘**’ 0.01 ‘*’ 0.05 ‘.’ 0.1 ‘ ’ 1.

**Table 17:** ANOVA table for an extension bulge posterior linear model.

Extension Bulge Posterior						
Var.	Df	Sum of Sq.	Mean Square	F Value	*p*-Value	Significance Code
Fiber910	1	0.29	0.29	17.9	4.54 × 10^−5^	***
Annulus_μ	1	0.74	0.74	45.6	4.90 × 10^−10^	***
Cartil_E	1	0.41	0.41	25.0	1.94 × 10^−6^	***
Residuals	124	2.02	0.02			

Significance codes: 0 ‘***’ 0.001 ‘**’ 0.01 ‘*’ 0.05 ‘.’ 0.1 ‘ ’ 1.

**Table 18:** ANOVA table for an extension stiffness linear model.

Extension Stiffness						
Var.	Df	Sum of Sq.	Mean Square	F Value	*p*-Value	Significance Code
Fiber910	1	13.2	13.2	20.4	1.45 × 10^−5^	***
Annulus_μ	1	8.6	8.6	13.3	3.88 × 10^−4^	***
Cartil_E	1	2.3	2.3	3.5	6.42 × 10^−2^	.
Residuals	124	80.4	0.6			

Significance codes: 0 ‘***’ 0.001 ‘**’ 0.01 ‘*’ 0.05 ‘.’ 0.1 ‘ ’ 1.

**Table 19:** ANOVA table for a lateral bending bulge lateral linear model.

Lateral Bending Bulge Lateral						
Var.	Df	Sum of Sq.	Mean Square	F Value	*p*-Value	Significance Code
C10	1	0.03	0.03	3.2	7.57 × 10^−2^	.
Annulus_μ	1	1.11	1.11	119.9	<2.0 × 10^−16^	***
Cartil_E	1	10.27	10.27	1112.5	<2.0 × 10^−16^	***
Cartil_μ	1	0.84	0.84	91.0	<2.0 × 10^−16^	***
Residuals	123	1.14	0.01			

Significance codes: 0 ‘***’ 0.001 ‘**’ 0.01 ‘*’ 0.05 ‘.’ 0.1 ‘ ’ 1.

**Table 20:** ANOVA table for a lateral bending bulge posterior linear model.

Lateral Bending Bulge Posterior						
Var.	Df	Sum of Sq.	Mean Square	F Value	*p*-Value	Significance Code
C10	1	0.26	0.26	53.7	2.84 × 10^−11^	***
C0	1	0.12	0.12	25.5	1.59 × 10^−6^	***
Annulus_E	1	0.06	0.06	11.8	8.21 × 10^−4^	***
Annulus_μ	1	2.34	2.34	478.3	<2.2 × 10^−16^	***
Cartil_E	1	17.71	17.71	3624.2	<2.2 × 10^−16^	***
Cartil_μ	1	0.23	0.23	47.1	3.06 × 10^−10^	***
Residuals	121	0.59	0.00			

Significance codes: 0 ‘***’ 0.001 ‘**’ 0.01 ‘*’ 0.05 ‘.’ 0.1 ‘ ’ 1.

**Table 21:** ANOVA table for a lateral bending stiffness linear model.

Lateral Bending Stiffness						
Var.	Df	Sum of Sq.	Mean Square	F Value	*p*-Value	Significance Code
C10	1	0.08	0.08	1.9	1.68 × 10^−1^	
Annulus_E	1	0.11	0.11	2.6	1.10 × 10^−1^	
Annulus_μ	1	11.63	11.63	265.9	<2.2 × 10^−16^	***
Cartil_E	1	56.69	56.69	1295.7	<2.2 × 10^−16^	***
Cartil_μ	1	1.15	1.15	26.3	1.11 × 10^−6^	***
Residuals	122	5.34	0.04			

Significance codes: 0 ‘***’ 0.001 ‘**’ 0.01 ‘*’ 0.05 ‘.’ 0.1 ‘ ’ 1.

**Table 22:** ANOVA table for a flexion bulge lateral linear model.

Flexion Bulge Lateral						
Var.	Df	Sum of Sq.	Mean Square	F Value	*p*-Value	Significance Code
C10	1	0.00012	0.00012	15.7	1.25 × 10^−4^	***
C0	1	0.00005	0.00005	6.2	1.38 × 10^−2^	*
Fiber12	1	0.00004	0.00004	5.0	2.74 × 10^−2^	*
Annulus_E	1	0.00002	0.00002	3.0	8.73 × 10^−2^	.
Annulus_μ	1	0.00313	0.00313	416.0	<2.2 × 10^−16^	***
Cartil_E	1	0.00316	0.00316	420.0	<2.2 × 10^−16^	***
Cartil_μ	1	0.00012	0.00012	15.5	1.42 × 10^−4^	***
Residuals	120	0.00090	0.00001			

Significance codes: 0 ‘***’ 0.001 ‘**’ 0.01 ‘*’ 0.05 ‘.’ 0.1 ‘ ’ 1.

**Table 23:** ANOVA table for a flexion bulge posterior linear model.

Flexion Bulge Posterior						
Var.	Df	Sum of Sq.	Mean Square	F Value	*p*-Value	Significance Code
C10	1	0.024	0.024	2130.8	<2.2 × 10^−16^	***
C0	1	0.011	0.011	949.1	<2.2 × 10^−16^	***
Annulus_E	1	0.003	0.003	264.3	<2.2 × 10^−16^	***
Annulus_μ	1	0.016	0.016	1429.9	<2.2 × 10^−16^	***
Cartil_μ	1	0.000	0.000	43.1	1.36 × 10^−9^	***
Residuals	122	0.001	0.000			

Significance codes: 0 ‘***’ 0.001 ‘**’ 0.01 ‘*’ 0.05 ‘.’ 0.1 ‘ ’ 1.

**Table 24:** ANOVA table for a flexion stiffness linear model.

Flexion Stiffness						
Var.	Df	Sum of Sq.	Mean Square	F Value	*p*-Value	Significance Code
C10	1	0.059	0.059	13.0	4.45 × 10^−4^	***
C0	1	0.027	0.027	5.9	1.68 × 10^−2^	*
Annulus_E	1	0.093	0.093	20.5	1.39 × 10^−5^	***
Annulus_μ	1	5.643	5.643	1243.7	<2.2 × 10^−16^	***
Cartil_E	1	5.706	5.706	1257.6	<2.2 × 10^−16^	***
Cartil_μ	1	0.044	0.044	9.6	2.42 × 10^−3^	**
Residuals	121	0.549	0.005			

Significance codes: 0 ‘***’ 0.001 ‘**’ 0.01 ‘*’ 0.05 ‘.’ 0.1 ‘ ’ 1.

**Table 25:** ANOVA table for a torsion stiffness linear model.

Torsion Stiffness						
Var.	Df	Sum of Sq.	Mean Square	F Value	*p*-Value	Significance Code
C10	1	0.032	0.032	21.0	1.13 × 10^−5^	***
C0	1	0.010	0.010	6.8	1.03 × 10^−2^	*
Fibra34	1	0.032	0.032	20.9	1.22 × 10^−5^	***
Fiber56	1	0.016	0.016	10.6	1.47 × 10^−3^	**
Fiber78	1	0.102	0.102	66.8	3.84 × 10^−13^	***
Annulus_E	1	1.056	1.056	691.1	<2.2 × 10^−16^	***
Annulus_μ	1	1.049	1.049	685.9	<2.2×10^−16^	***
Cartil_E	1	5.539	5.539	3623.7	<2.2 × 10^−16^	***
Cartil_μ	1	0.087	0.087	56.9	1.04 × 10^−11^	***
Residuals	118	0.180	0.002			

Significance codes: 0 ‘***’ 0.001 ‘**’ 0.01 ‘*’ 0.05 ‘.’ 0.1 ‘ ’ 1.

**Table 26:** Results of the predicted error criteria using the regression models.

**Errors and Correlations**	**Comp_BulgeA**	**Comp_BulgeL**	**Comp_BulgeP**	**Comp_Stiff**	**Shear_Stiff**
Correlation	99.189	96.861	99.180	98.784	99.636
MAE	3.384	7.353	2.839	5.166	1.740
RMSE	3.710	7.786	3.433	5.228	2.190
	**Exte_BulgeL**	**Exte_BulgeP**	**Exte_Stiff**	**LBend_BulgeL**	**LBen_BulgeP**
Correlation	84.457	64.532	48.035	95.663	98.603
MAE	11.141	13.241	13.973	8.023	4.176
RMSE	17.393	19.936	21.812	9.483	5.135
	**LBend_Stiff**	**Flex_BulgeL**	**Flex_BulgeP**	**Flex_Stiff**	**Tors_Stiff**
Correlation	96.376	93.817	98.757	97.709	98.881
MAE	8.956	8.864	3.203	6.036	3.257
RMSE	9.072	9.840	3.607	6.441	3.997

**Table 27:** Results of the predicted error criteria using the regression models.

**Errors and Correlations**	**Comp_BulgeA**	**Comp_BulgeL**	**Comp_BulgeP**	**Comp_Stiff**	**Shear_Stiff**
Correlation	98.801	95.648	95.429	97.603	94.763
MAE	5.998	12.781	8.063	4.835	6.320
RMSE	6.792	14.106	9.167	6.031	7.802
	**Exte_BulgeL**	**Exte_BulgeP**	**Exte_Stiff**	**LBend_BulgeL**	**LBend_BulgeP**
Correlation	92.228	82.880	69.068	88.825	91.281
MAE	6.595	5.537	9.807	8.575	8.315
RMSE	7.574	6.884	11.204	11.663	10.359
	**LBend_Stiff**	**Flex_BulgeL**	**Flex_BulgeP**	**Flex_Stiff**	**Tors_Stiff**
Correlation	87.637	92.933	96.930	94.031	64.698
MAE	9.122	6.981	2.8642	5.594	10.067
RMSE	11.125	8.152	3.193	6.883	19.505

**Table 28:** The first criterion considered inputs and outputs that were considered equally important.

Var.	Goal	Value	Desirability
C10	inRange → 0.125	0.102	1.000
C0	inRange → 0.03	0.015	1.000
Fiber12	inRange → 532.5	518.133	1.000
Fiber34	inRange → 509	500.083	1.000
Fiber56	inRange → 479	517.692	1.000
Fiber78	inRange → 431.5	463.054	1.000
Fiber910	inRange → 384	366.794	1.000
Annulus_E	inRange → 4.1	3.951	1.000
Annulus_μ	inRange → 0.35	0.201	1.000
Cartil_E	inRange → 39	42.121	1.000
Cartil_μ	inRange → 0.35	0.430	1.000
Comp_bulgeA	target → 0.5	0.265	0.262
Comp_bulgeL	target → 0.35	0.097	0.244
Comp_bulgeP	target → 0.75	0.650	0.651
Comp_stiff	target → 810	826.143	0.983
Shear_stiff	target → 300	298.124	0.964
Exte_bulgeL	target → 0.1	0.100	1.000
Exte_bulgeP	target → 0.24	0.490	0.711
Exte_stiff	target → 1.53	2.167	0.867
LBend_bulgeL	target → 2.11	1.235	0.534
LBend_bulgeP	target → 1.13	1.459	0.792
LBend_stiff	target → 2	1.465	0.634
Flex_bulgeL	target → 0.07	0.074	0.873
Flex_bulgeP	target → 0.73	0.375	0.381
Flex_stiff	target → 1.18	1.357	0.880
Tors_stiff	target → 2.1	3.401	0.451
Overall Desirability	0.625		

**Table 29:** The second criterion considered: setting the target of the FE model parameters based only on stiffness.

Var.	Goal	Value	Desirability
C10	inRange → 0.125	0.105	1.000
C0	inRange → 0.03	0.015	1.000
Fiber12	inRange → 532.5	541.867	1.000
Fiber34	inRange → 509	500.123	1.000
Fiber56	inRange → 479	458.643	1.000
Fiber78	inRange → 431.5	396.291	1.000
Fiber91	inRange → 384	421.320	1.000
Annulus_E	inRange → 4.1	3.952205	1.000
Annulus_μ	inRange → 0.35	0.2269	1.000
Cartil_E	inRange → 39	45.575	1.000
Cartil_μ	inRange → 0.35	0.2756	1.000
Comp_bulgeA	inRange → 0.5	0.262	1.000
Comp_bulgeL	inRange → 0.35	0.089	1.000
Comp_bulgeP	inRange → 0.75	0.628	1.000
Comp_stiff	target → 810	900.147	0.907
Shear_stiff	target → 300	300.000	1.000
Exte_bulgeL	inRange → 0.1	0.105	1.000
Exte_bulgeP	inRange → 0.24	0.605	1.000
Exte_stiff	target → 1.53	1.530	0.999
LBend_bulgeL	inRange → 2.11	0.895	1.000
LBend_bulgeP	inRange → 1.13	1.270	1.000
LBend_stiff	target → 2	1.984	0.989
Flex_bulgeL	inRange → 0.07	0.076	1.000
Flex_bulgeP	inRange → 0.73	0.363	1.000
Flex_stiff	target → 1.18	1.518	0.772
Tors_stiff	target → 2.1	3.456	0.428
Overall Desirability	0.817		

**Table 30:** The third criterion considered: setting the target of the FE model parameters based only on the bulges.

Var.	Goal	Value	Desirability
C10	inRange → 0.125	0.102	1.000
C0	inRange → 0.03	0.015	1.000
Fiber12	inRange → 532.5	559.341	1.000
Fiber34	inRange → 509	512.790	1.000
Fiber56	inRange → 479	443.243	1.000
Fiber78	inRange → 431.5	396.348	1.000
Fiber91	inRange → 384	348.282	1.000
Annulus_E	inRange → 4.1	3.951	1.000
Annulus_μ	inRange → 0.35	0.214	1.000
Cartil_E	inRange → 39	36.933	1.000
Cartil_μ	inRange → 0.35	0.429	1.000
Comp_bulgeA	target → 0.5	0.277	0.298
Comp_bulgeL	target → 0.35	0.101	0.257
Comp_bulgeP	target → 0.75	0.673	0.730
Comp_stiff	inRange → 810	821.981	1.000
Shear_stiff	inRange → 300	287.011	1.000
Exte_bulgeL	target → 0.1	0.103	0.947
Exte_bulgeP	target → 0.24	0.481	0.722
Exte_stiff	inRange → 1.53	2.403	1.000
LBend_bulgeL	Target → 2.11	1.314	0.576
LBend_bulgeP	target → 1.13	1.595	0.706
LBend_stiff	inRange → 2	1.288	1.000
Flex_bulgeL	target → 0.07	0.075	0.846
Flex_bulgeP	target → 0.73	0.373	0.378
Flex_stiff	inRange → 1.18	1.315	1.000
Tors_stiff	inRange → 2.1	3.311	1.000
Overall Desirability	0.554		

**Table 31:** Comparison of the results of the regression models; FEM and the experimental values.

Parameters	Criteria 1	Criteria 2	Criteria 3	Experiments	Error
	FEM	FEM	FEM	Standard Test	Normalized MAE
Comp_bulgeA	0.266	0.262	0.269	0.50	0.469
Comp_bulgeL	0.096	0.090	0.095	0.35	0.732
Comp_bulgeP	0.624	0.602	0.625	0.75	0.177
Comp_stiff	915.640	944.360	922.740	810	0.125
Shear_stiff	302.925	304.920	299.090	300	0.010
Exte_bulgeL	0.106	0.099	0.106	0.10	0.041
Exte_bulgeP	0.527	0.559	0.538	0.24	0.539
Exte_stiff	1.634	1.678	1.645	1.53	0.073
LBend_bulgeL	0.997	0.871	0.977	2.11	0.551
LBend_bulgeP	1.282	1.173	1.263	1.13	0.085
LBend_stiff	1.489	2.175	1.493	2.00	0.183
Flex_bulgeL	0.077	0.080	0.076	0.07	0.096
Flex_bulgeP	0.380	0.373	0.373	0.73	0.486
Flex_stiff	1.488	1.524	1.500	1.18	0.213
Tors_stiff	3.550	3.549	3.506	2.10	0.404
Normalized MAE	0.2782	0.2795	0.2788		

## List of Symbols/Nomenclature

**MAE**	**Mean Absolute Error**	
C10	Mooney-Rivlin parameter for annulus ground substance	
C0	Mooney-Rivlin parameter for annulus ground substance	
Annulus_E	Young’s modulus for the annulus ground substance	(MPa)
Annulus_μ	Poisson’s modulus for the annulus ground substance	
Fiber12	Collagen fibers layer 1	(mm^2^)
Fiber34	Collagen fibers layer 2	(mm^2^)
Fiber56	Collagen fibers layer 3	(mm^2^)
Fiber78	Collagen fibers layer 4	(mm^2^)
Fiber910	Collagen fibers layer 5	(mm^2^)
Cartil_E	Young’s modulus for the endplate cartilage	(MPa)
Cartil_μ	Poisson’s modulus for the endplate cartilage	-
Comp_bulgeA	Bulge Anterior for the Compression Test	(mm)
Comp_bulgeL	Bulge Lateral for the Compression Test	(mm)
Comp_bulgeP	Bulge Posterior for the Compression Test	(mm)
Comp_stiff	Stiffness for the Compression Test	(N/mm)
Shear_stiff	Stiffness for the Shear Test	(N/mm)
Exte_bulgeL	Bulge Lateral for the Extension Test	(mm)
Exte_bulgeP	Bulge Posterior for the Extension Test	(mm)
Exte_stiff	Stiffness for the Extension Test	(Nm/°)
LBend_bulgeL	Bulge Lateral for the Lateral Bending Test	(mm)
LBend_bulgeP	Bulge Posterior for the Lateral Bending Test	(mm)
LBend_stiff	Stiffness for the Lateral Bending Test	(Nm/°)
Flex_bulgeL	Bulge Lateral for the Flexion Test	(mm)
Flex_bulgeP	Bulge Posterior for the Flexion Test	(mm)
Flex_stiff	Stiffness for the Flexion Test	(Nm/°)
Tors_stiff	Stiffness for the Torsion Test	(Nm/°)
